# The effect and mechanism of total alkaloids of *Fritillariae Pallidiflorae* Bulbus in alleviating pulmonary fibrosis

**DOI:** 10.3389/fphar.2026.1849543

**Published:** 2026-07-08

**Authors:** Mingxin Pai, Zhongzhi Qi, Xinyu Zhou, Qian Shi, Yexin Wu, Mengru Teng, Yuanyuan Chen, Wai Ming Tse, Kathy Wai Gaun Tse, Mingji Cuomu, Bengui Ye

**Affiliations:** 1 Nuclear Medicine Department of West China Hospital of Sichuan University, Chengdu, China; 2 Key Laboratory of Drug Targeting and Drug Delivery System of the Education Ministry, Sichuan Engineering Laboratory for Plant-Sourced Drug and Sichuan Research Center for Drug Precision Industrial Technology, West China School of Pharmacy Sichuan University, Chengdu, China; 3 Nin Jiom Medicine Manufactory (H.K.) Limited, Chengdu, China; 4 Department of Tibetan Medicine, Xizang University of Tibetan Medicine, Lhasa, China; 5 Medical College of Xizang University, Lhasa, China

**Keywords:** alkaloids, *Fritillariae Pallidiflorae* Bulbus, imperialine, omics, pulmonary fibrosis

## Abstract

**Background:**

Pulmonary fibrosis (PF) is a chronic progressive disease characterized by inflammatory response and fibroblast activation. This process leads to abnormal deposition of extracellular matrix (ECM). However, the existing clinical treatment methods have significant side effects, poor patient compliance, and limited efficacy. *Fritillariae Pallidiflorae* Bulbus (BFP) refers to the desiccated bulb of *Fritillaria pallidiflora* Schrenk, employed for managing persistent pulmonary conditions, with alkaloids as its main active compounds.

**Methods:**

This study examined the efficacy and mechanism of action of the total alkaloids of *Fritillariae Pallidiflorae* Bulbus (BFP-TA) in treating PF. The material composition of BFP-TA was assessed through High-Performance Liquid Chromatography with Evaporative Light-Scattering Detector (HPLC-ELSD), High-Performance Liquid Chromatography-Quadrupole-Time of Flight Mass Spectrometry (HPLC-Q-TOF-MS/MS). The potential molecular mechanisms of BFP-TA were explored through multi-omics analysis and molecular biology techniques in a rat model of PF induced by bleomycin (BLM). The effects and mechanisms of the medicated plasma components of BFP-TA were validated by integrating blood component analysis with a TGF-β1-induced A549 cell model.

**Results:**

In BFP-TA, 182 components were detected, comprising 26 alkaloids from genus *Fritillariae*, with 9 of these alkaloids representing 63.643% of the total. BFP-TA has been shown to improve the progression of PF in rats by reducing inflammation and the PF phenotype. Multi-omics analysis suggests that BFP-TA may alleviate PF by regulating Rap1/ERK signaling pathway. It may also be related to biological processes such as cell migration and cell adhesion. The primary blood-entering component of BFP-TA is imperialine, which exhibits a significant anti-PF effect.

**Conclusion:**

This study explored the therapeutic effect of BFP-TA on PF. The results showed that BFP-TA may regulate the Rap1/ERK signaling pathway to reduce the expression of pro-inflammatory factors and collagen deposition, thereby improving lung injury and fibrosis. The imperialine in BFP-TA also contributes to the anti-fibrosis ability.

## Graphical Abstract

**Figure:**
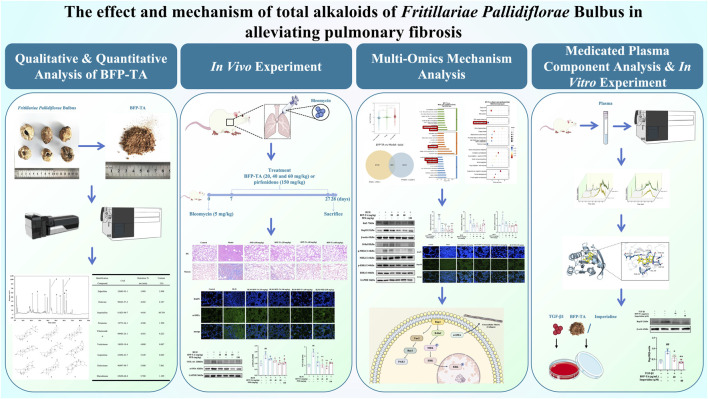

## 1 Introduction

Pulmonary Fibrosis (PF) is an irreversible, chronic, and progressive interstitial lung disease. It involves ECM deposition, leading to lung structure remodeling, which ultimately impairs lung compliance, causing respiratory failure and mortality (Savin et al., 2022; Zheng H. et al., 2025). Due to limited therapeutic options, the life expectancy of patients after diagnosis is usually three to 5 years. Idiopathic pulmonary fibrosis (IPF) constitutes 20%-30% of PF cases and is the most prevalent fatal subtype, with a 5-year survival rate below 50%, contributing significantly to mortality from chronic lung conditions (Spagnolo et al., 2022). The exact pathogenesis of PF remains unclear. Current clinical management relies on pharmacologic and nonpharmacologic therapies. Non-pharmacologic treatments include pulmonary rehabilitation and lung transplantation. However, indications for pulmonary rehabilitation may lead to decreased lung pathophysiology and quality of life, whereas lung transplantation is associated with multiple risks such as bleeding, infection and rejection with high risk and poor prognosis (Schwarz et al., 2021). Pharmacologic therapy relies mainly on pirfenidone (PFD) and nintedanib, PFD and nintedanib are the two core drugs for the treatment of idiopathic pulmonary fibrosis (IPF), but both drugs have significant hepatotoxicity, while their side effects constrain the overall prognosis and clinical utility of PF (Wells et al., 2020). The investigation of novel molecular targets and the creation of therapeutic drugs have taken center stage in contemporary research due to the intricacy of the pathological processes of PF and the shortcomings of the present treatment methods.

The pathogenesis of PF is not driven by a single factor, but rather by a multilayered interaction of genetic predisposition, environmental exposures, acquired injury, and immune imbalance, including smoking, viral infections, gastroesophageal reflux, aging, environmental stimuli, and medications. Together, these factors damage the alveolar epithelium, triggering oxidative stress, inflammation and apoptosis. Subsequently, the damaged lung epithelium releases cytokines and growth factors, triggering pro-fibrotic signaling pathways, which activate lung fibroblasts and differentiate them into myofibroblasts. Consequently, extracellular matrix (ECM) is deposited, resulting in the development of fibrotic scarring in the lung parenchyma, leading to the pathological condition of PF (Watanabe et al., 2021; Borok et al., 2020; Fathimath Muneesa et al., 2021; Kishore and Petrek, 2021).

The unique diagnostic and treatment framework of traditional Chinese medicine provides significant advantages. The principles of evidence-based and holistic care are firmly established in clinical practice (Hao et al., 2024; Wang et al., 2023). PF in the *Synopsis of the Golden Chamber* is described as ‘lung impediment’ or ‘lung wilting’, with syndrome types such as lung dryness, Qi deficiency, and phlegm-stasis (Du et al., 2024). As research on PF treatment mechanisms advances, certain traditional Chinese medicines have demonstrated diverse mechanisms of action targeting multiple pathways. The *Compendium of Materia Medica* documents that *Fritillaria* is effective in managing conditions such as ‘lung wilting’,‘lung impediment’, ‘cough’, ‘lung distension’, ‘asthma syndrome’ and so on. BFP is the desiccated bulb of *Fritillaria pallidiflora* Schrenk, a plant of the genus *Fritillaria* (State Pharmacopoeia Committee Chinese Pharmacopoeia, 2025). It clears heat, moistens the lungs, transforms phlegm, and alleviates cough. It addresses dry cough due to lung heat, dry cough with minimal sputum, labor cough from Yin deficiency, and cough with blood. Research indicates that BFP effectively reduces phlegm and cough, easing wheezing symptoms (Wu et al., 2022), and BFP effectively treats pulmonary fibrosis and inflammation (Wang et al., 2025; Jiao et al., 2020). The main active component of BFP is isosteroidal alkaloids (Xu et al., 2019). Studies has indicated that the primary bioactive compounds in BFP, including imperialine and peiminine, exhibit effective therapeutic properties against pulmonary inflammation and PF (Lin et al., 2019; Guo et al., 2016). Previous studies have demonstrated that total alkaloids in *Fritillaria* have the potential to counteract a variety of chronic lung diseases (Wang et al., 2024a; Wang et al., 2024b). In conclusion, experimental research and traditional Chinese medicine theory indicate the significant potential of BFP-TA in treating PF. Additional research is needed to clarify the specific mechanism of BFP-TA in addressing PF.

Transcriptomics and proteomics methods were combined in this study, along with bioinformatics analysis, molecular docking techniques, analysis of incoming blood components, and *in vivo* and *in vitro* experiments. The aim was to systematically investigate the active components of BFP-TA and the effects and mechanisms of its PF-alleviating action. Transcriptomics and proteomics analyses were integrated to investigate crucial biological processes and signaling pathways. Blood component analysis investigated the main constituents of BFP-TA in the bloodstream. Molecular docking validated the binding affinity of the main active compounds with key target proteins, confirming the molecular mechanism of action.

## 2 Materials and methods

### 2.1 Materials

ELISA kits for interleukin (IL)-4 (JYM0647Ra), IL-10 (JYM0651Ra), tumor necrosis factor α (TNF-α) (JYM0635Ra), and interferon γ (IFN-γ) (JYM0654Ra) were acquired from Wuhan Genemei Biotechnology Co., Ltd. Hydroxyproline kits (A030-2-1) were obtained from Nanjing Jiancheng Biotechnology Co., Ltd., and bleomycin (BLM) (B981763) from Shanghai Maclean Biotechnology Co., Ltd. The PrimeScript™ RT reagent Kit gDNA Eraser (RR047A) was procured from Takara. The SYBRPRIME PCR KIT (Fast HS) (BG0014) was purchased from Chongqing Baoguang Biotechnology Co., Ltd., and Recombinant Human TGF-β1 (100-21) was purchased from PeproTech. α-SMA (ab124964), purchased from Abcam. Rap1B (10840-1-AP), MEK1/2 (11049-1-AP), purchased from Proteintech. B-Raf (AF6170), p-MEK1/2 (AF8035), ERK1/2 (AF0155), GAPDH (AF7021), purchased from Affinity Biosciences. p-ERK1/2 (AP0485), purchased from Abclonal. β-actin (#4967), COL1A1 (#84336), purchased from Cell Signaling Technology.

### 2.2 Preparation and analysis of BFP-TA

BFP was gathered in Gongliu County, Xinjiang Uygur Autonomous Region, China. It was recognized as the desiccated bulb of *F. pallidiflora* Schrenk, a plant of the genus *Fritillaria* in the family Liliaceae by the Department of Pharmacognosy at West China School of Pharmacy, Sichuan University. The voucher specimen (V2023110801) is preserved in the Pharmacognosy Laboratory of West China School of Pharmacy, Sichuan University.

The desiccated BFP was pulverized, weighed, and sieved, then heated under reflux twice with 70% ethanol. The filtrate underwent concentration under reduced pressure and desiccation to yield the BFP ethanol extract. Subsequently, the extract underwent further purification through cation exchange resin and macroporous adsorption to produce BFP-TA. The purified alkaloid underwent qualitative and quantitative analysis using High-Performance Liquid Chromatography with Evaporative Light-Scattering Detector, High-Performance Liquid Chromatography-Quadrupole-Time of Flight Mass Spectrometry, and liquid chromatography-tandem mass spectrometry. Additional details on the extraction, purification, and analytical methods for BFP-TA are available in the Supplementary Material.

### 2.3 Animals and experimental design

Six-week-old male Sprague-Dawley (SD) rats weighing 180-220 g were obtained from Beijing Sibeifu Biotechnology Co., Ltd. (SYXK (Chuan) 2018-113) and housed in the Animal Centre of West China School of Pharmacy, Sichuan University. The rats were maintained in a specific pathogen-free (SPF) facility with standard conditions: temperature (20 °C–24 °C), humidity (60%-70%), and a 12-h light-dark cycle. Animal procedures followed the National Institute of Health guidelines for laboratory animal care and were authorized by the Sichuan University Ethics Committee (approval number: K2024036).

After a 7-day acclimation, SD rats were randomly assigned to 6 groups (n = 6): control (solvent blank), model (BLM, 5 mg/kg), low-dose (BFP-TA, 20 mg/kg/d), medium-dose (BFP-TA, 40 mg/kg/d), high-dose (BFP-TA, 60 mg/kg/d), and PFD (PFD, 150 mg/kg/d) groups. The rats in the experiment were anesthetized with a 1% sodium pentobarbital solution injected intraperitoneally. After achieving anesthesia, the rats were placed supine on the experimental table and their tongues were gently extended. The neck area was then depilated and disinfected using 75% ethanol. Sterile techniques were followed to perform a midline neck incision for trachea exposure through blunt dissection. The trachea was punctured at a 45-degree angle using a syringe, and a single dose BLM solution immediately was injected. Subjects in the control group were administered the same volume of physiological saline solution.

One week post-model establishment, all groups of animals began daily gavage treatment. Throughout the experiment, the physiological condition and survival status of the experimental animals were observed and recorded daily, while weight changes were measured every 72 h. After 3 weeks intervention period, general anesthesia was performed by intraperitoneal injection of 1% pentobarbital sodium solution. Subsequently, serum samples were collected in turn and lung organs were completely removed. The wet weight of lung tissue was measured after removing residual blood with pre-cooled normal saline. In a standard laboratory environment, the tissue from the right lung was promptly moved to −80 °C for further analysis. The tissue from the left lung was subjected to fixation using a 4% paraformaldehyde solution.

### 2.4 Pathological section staining and immunofluorescence

Hematoxylin-Eosin (HE) staining, Masson staining, and immunofluorescence staining were performed on lung tissue by Chengdu Lilai. Specifically, the left lung tissue was first fixed in 4% paraformaldehyde solution for a duration of 48 h. Subsequently, the tissue was embedded in paraffin wax and sectioned into slices with a thickness of 5 μm. These sections underwent staining with HE and Masson for assessing lung histopathological alterations, and images were taken using an optical microscope. Following this step, the tissue sections underwent staining procedures utilizing DAPI dye, α-smooth muscle actin (α-SMA) antibody, and Rap1B antibody. This was performed to assess the localization and expression of α-SMA and Rap1B within the lung tissue. Subsequently, fluorescent inverted microscopy was employed to capture the images.

### 2.5 Enzyme-linked immunosorbent assay (ELISA)

A blood sample of 5 mL was taken from the heart and centrifuged at 6,000 rpm for 20 min at 4 °C using a refrigerated centrifuge (TGL-16S, Sichuan Shuke Instrument Co., Ltd., Chengdu, China). ELISA kits from Wuhan Jiyinmei Biotechnology Co., Ltd., (Wuhan, China) were employed to evaluate TNF-α, IFN-γ, IL-4, and IL-10 levels in rat serum. A 50 mg fresh right lung tissue sample was homogenized in 0.45 mL of cold PBS. The homogenate was then centrifuged at 6,000 rpm for 15 min at 4 °C, and the resulting supernatant was aliquoted. Following the guidelines provided in the kit, the analyses were carried out. Subsequently, the optical density values were measured at a wavelength of 450 nm utilizing a microplate reader. The concentrations of cytokines were quantified in units of picograms per milliliter.

### 2.6 Determination of hydroxyproline (HYP) content

Alkaline hydrolysis was employed to quantify hydroxyproline (HYP) levels in lung tissue. First, precisely 50 mg of fresh right lung tissue was weighed out and transferred into a test tube. Subsequently, 1 mL of hydrolysis solution was added into the tube, which was then placed in a boiling water bath and heated for 20 min to promote the hydrolysis process. Once the hydrolyzed solution had cooled to room temperature, its pH was adjusted to the range of approximately 6.0–6.8. The lysate underwent centrifugation at a speed of 7,000 r/min for a duration of 10 min under room temperature conditions. Following this, 1 mL of the supernatant obtained from the centrifugation step was carefully pipetted into a fresh test tube. The experimental procedures were carried out in strict accordance with the instructions provided in the HYP assay kit (Product No.: #A030-2-1; Nanjing Jianchen Bioengineering Institute, Nanjing, Jiangsu, China). Next, the absorbance of each sample was measured at a wavelength of 550 nm using a UV spectrophotometer (TU-1810SPC, Beijing Purkinje General Instrument, Beijing, China). Based on the standard curve, the HYP concentration in each sample was subsequently calculated.

### 2.7 Western blotting

Employing a lysis buffer supplemented with protease and phosphatase inhibitors (Signalway Antibody, USA) as well as phenylmethylsulfonyl fluoride (PMSF, Beyotime, Shanghai, China), the proteins underwent purification via centrifugation at 4 °C for a duration of 30 min. Subsequently, the obtained protein sample underwent centrifugation at a speed of 12,000 × *g* for a duration of 15 min, with the temperature maintained at 4 °C. The protein concentration was assessed utilizing the Bicinchoninic Acid (BCA) Protein Assay Kit (Beyotime, Shanghai, China). Following this, an amount of 40 μg protein was loaded onto a 7.5% sodium dodecyl sulfate-polyacrylamide gel electrophoresis (7.5% SDS-PAGE gel, #PG111, Epizyme, Shanghai, China) system for separation. After that, the separated proteins were transferred onto a polyvinylidene fluoride (PVDF) membrane (Millipore, USA). The membrane was incubated in a Tris-buffered saline Tween-20 (TBST) solution containing 5% (wt/vol) nonfat dry milk for 1 h at ambient temperature. For antibody detection, the membrane was then probed with primary antibodies specific for α-SMA, COL1A1, Rap1B, B-Raf, MEK1/2, phosphorylated MEK1/2 (p-MEK1/2), ERK1/2, phosphorylated ERK1/2 (p-ERK1/2), β-actin, and GAPDH overnight at 4 °C, with β-actin and GAPDH serving as internal reference controls. After rinsing the membrane with TBST buffer, the samples were incubated with horseradish peroxidase (HRP)-conjugated secondary antibodies at room temperature for 1 h. Then, protein bands were visualized using an enhanced chemiluminescence detection system.

### 2.8 Real-time quantitative PCR (qRT-PCR)

Lung issue were processed to extract total RNA using TRIzol reagent protocol. The Nanodrop 2000 spectrophotometer assessed RNA quantity and purity, with A260/280 ratios ranging from 1.8 to 2.0. The gDNA Eraser in the PrimeScrip™ RT Reagent Kit eliminated DNA to facilitate cDNA synthesis via reverse transcription. Real-time PCR was carried out on the QuantStudio system using the SYBR Prime PCR Kit. The PCR procedure commenced with an initial incubation at 95 °C for a duration of 2 min. Whereafter, it underwent 40 amplification cycles, each consisting of denaturation at 95 °C for 5 s, annealing at 60 °C for 30 s, and an extension phase also at 95 °C for 5 s. To quantify gene expression levels, the 2^−ΔΔCt^ method was employed, utilizing GAPDH as the reference gene for normalization. Primers, including GAPDH (No: B661304), were sourced from Sangon Biotech, with detailed sequences in Supplementary Table S1.

### 2.9 Proteomics

The samples were frozen deeply and ground thoroughly. Next, lysis buffer, phosphatase inhibitors, and protease inhibitors were added sequentially. Grinding using a cold grinder was followed by centrifugation to eliminate the supernatant, which was subsequently centrifuged to obtain the complete protein solution. The determination of protein concentration was carried out by employing the BCA assay, following the guidelines outlined in the kit manual. The protein amount extracted from each sample was adjusted based on the measured concentration and diluted with lysis buffer. DTT was added in an ice bath for rapid cooling, followed by the addition of iodoacetamide to the reaction system for incubation in darkness. Proteins were precipitated using organic solvent, then centrifuged to collect the protein precipitate, discarding the supernatant. The precipitate was dissolved in a 50 mM NH4HCO3 solution, and TPCK was added to modify trypsin enzymatic hydrolysis. Phosphoric acid was added to adjust the solution’s pH to around 3 to halt the enzymatic hydrolysis reaction. The column was activated with methanol, equilibrated with the equilibration solution, and the sample was loaded onto the column. The column was washed with an aqueous solution containing 0.1% formic acid. The peptides were eluted using a 50% acetonitrile-water solution and then evaporated through vacuum drying. In the DIA liquid phase elution process, a gradient with specific conditions was employed. Buffer A was composed of an aqueous solution with 0.1% formic acid, while Buffer B comprised acetonitrile containing 0.1% formic acid. Following peptide separation, mass spectrometry analysis was conducted in a data-independent manner utilizing the Bruker timsTOF HT mass spectrometer. The parameters for the mass spectrometry analysis were set as follows: a capillary voltage of 1.6 kV, a drying temperature maintained at 180 °C, a consistent gas flow rate of 3.0 L per minute, a scan range spanning from 300 to 1500 m/z, an ion mobility range of 0.75 to 1.3, and collision energy adjusted between 20 and 59 eV. DIA protein quantification was performed with DIA-NN software. Pro DIA proteomics was performed by Shanghai OE Biotech Co., Ltd.

### 2.10 Cell culture and treatment

A549 human alveolar basal epithelial cells, which originate from lung carcinoma and were sourced from the American Type Culture Collection (USA), were subjected to thawing at 37 °C subsequent to their removal from liquid nitrogen storage. Upon thawing, the cells were placed in a 5 mL centrifuge tube, followed by the addition of 2 mL of complete medium. Gentle shaking was performed to ensure uniform distribution, and subsequent centrifugation at 1,000 r/min for 3 min led to the removal of the supernatant. The cell pellet was then resuspended in 1 mL of complete medium. The resuspended cells were transferred to a culture dish, and additional culture medium was added to reach a total volume of 5 mL. After gentle mixing, the dish was placed in a 37 °C, 5% CO2 incubator for cultivation. Following trypsin digestion, 10 μL of the cell suspension was collected for cell counting, and the dilution factor was calculated before plating in a 6-well plate. The cells were incubated until they reached 80% confluence and were then starved overnight. Drug administration was conducted according to the following protocol: Control group received basal medium plus DMSO; the Model group consisted of TGF-β1 (PeproTech, USA)-induced A549 cells. The cells were incubated for 48 h before being utilized for subsequent experiments.

### 2.11 Medicated plasma component analysis

A total of 5 ml of blood samples were collected from SD rats into tubes containing sodium heparin 30 minutes prior to oral administration of BFP-TA, and 15, 30, 60, 120, 240, 480, 600 and 1440 minutes after oral administration of BFP-TA. Blood specimens were subjected to centrifugation at a speed of 3,500 revolutions per minute for a duration of 15 min at a temperature of 4 °C, and subsequently stored at −80 °C. For each sampling time point, 100 μL of the obtained plasma was pooled together, followed by protein precipitation using a triple volume of acetonitrile. The resultant mixture underwent centrifugation at 12,000 revolutions per minute for 2 min. The supernatant was then evaporated to dryness using liquid nitrogen, redissolved in methanol, and finally subjected to analysis via HPLC-Q-TOF-MS/MS.

### 2.12 Molecular docking

The molecular structure files for the target compounds were obtained from the PubChem database. The corresponding receptor protein structure was then obtained from the PDB database. Following this, AutoDockTools was utilized to optimize the receptor protein structure, which included the addition of hydrogen atoms and charge balancing. The processed receptor structure and the ligand small molecule were then converted into pdbqt file format to meet the requirements for molecular docking calculations. During the molecular docking phase, AutoDock Vina software (v 1.1.2) facilitated the docking of the receptor protein with the ligand small molecule. Upon completion of the docking process, the PLIP tool was employed for a detailed analysis of the docking results.

### 2.13 Statistical analysis

The statistical analyses were conducted using GraphPad Prism 9 (GraphPad Software Inc., USA). Data are expressed as the mean ± standard deviation (SD). Comparisons among groups were performed using one-way analysis of variance (ANOVA), followed by Tukey’s *post hoc* test. A *P* < 0.05 was considered statistically significant.

## 3 Results

### 3.1 Identification of chemical components in BFP-TA by HPLC-Q-TOF-MS/MS

**FIGURE 1:**
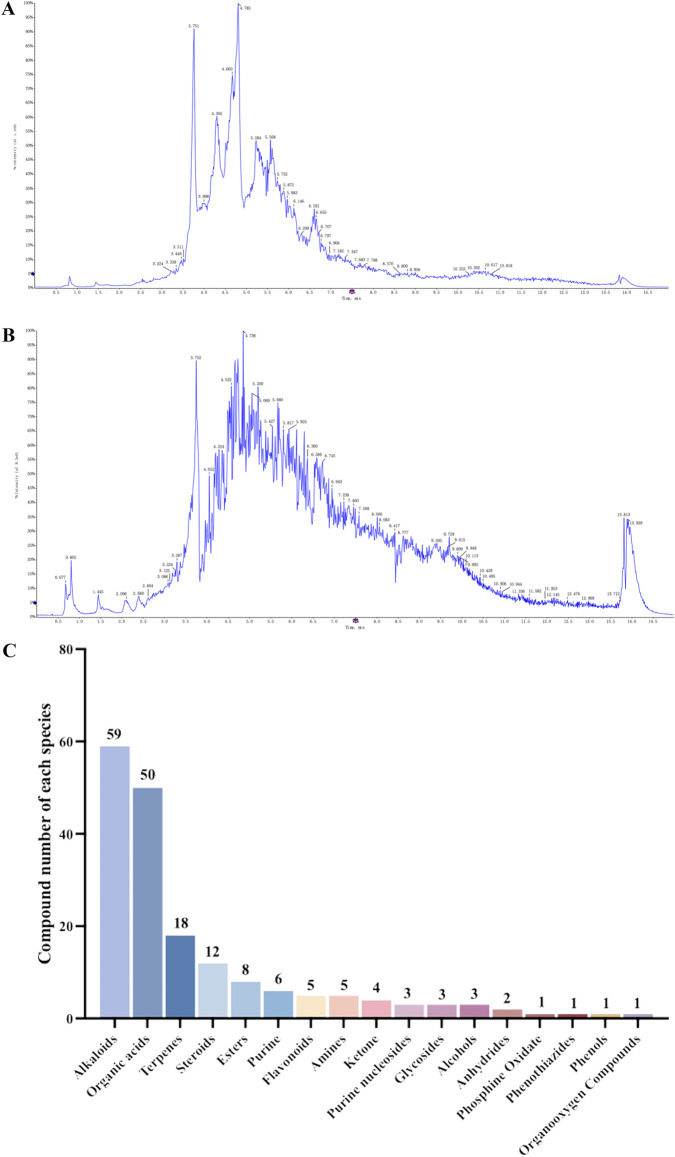
Chemical constituents of BFP-TA. **(A)** positive ion flow diagram, **(B)** negative ion flow diagram, **(C)** types and quantities of compounds in BFP-TA.

The chemical compounents of BFP-TA was determined by HPLC-Q-TOF-MS/MS. An extensive examination revealed 17 types of compounds in BFP-TA. Additionally, HPLC-Q-TOF-MS/MS detected 182 chemical components in BFP-TA (Figure 1; Supplementary Table S2). There were 59 alkaloids in BFP-TA, of which 26 of these compounds recognized as alkaloids unique to the genus *Fritillaria*.

### 3.2 Quantitative study of 9 alkaloids in BFP-TA

**FIGURE 2:**
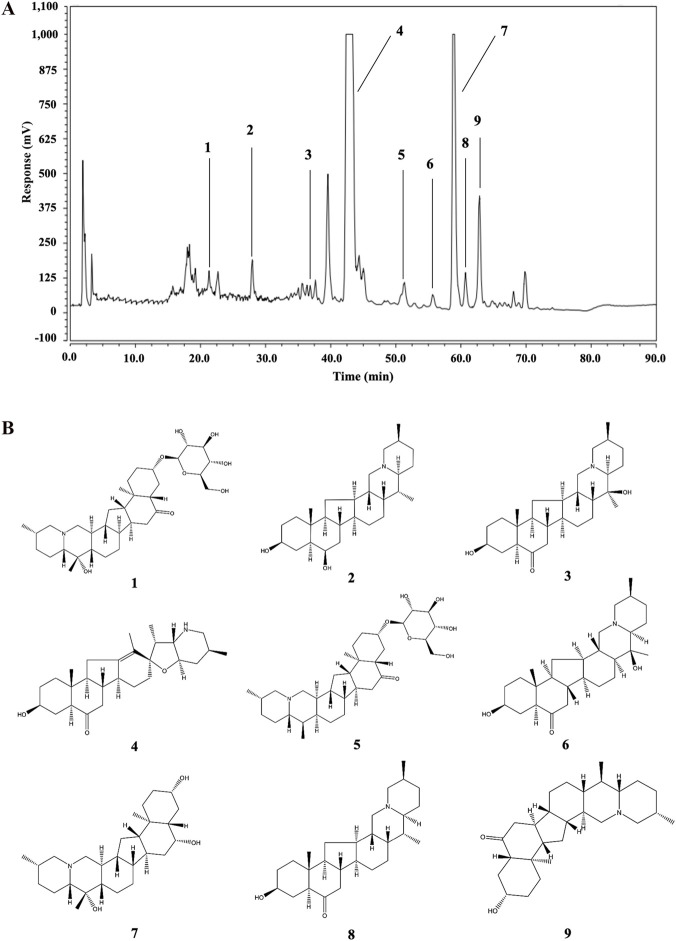
HPLC-ELSD analysis of BFP-TA. **(A)** HPLC-ELSD analysis in BFP-TA, **(B)** Structural formula of 9 alkaloids in BFP-TA.

The study compared and analyzed the retention times of standard samples of alkaloids isolated in the laboratory with those of BFP-TA to identify the alkaloid types present. Preliminary qualitative analysis of nine alkaloids enriched in BFP-TA was performed using HPLC-ELSD technology (Figure 2A). Subsequently, the 9 alkaloids were quantitatively studied using LC-MS/MS with the external standard method (Figure 2B; Supplementary Tables S3, S4). The cumulative percentage of these 9 alkaloids amounts to 63.643%.

### 3.3 BFP-TA alleviates BLM-induced lung injury

**FIGURE 3:**
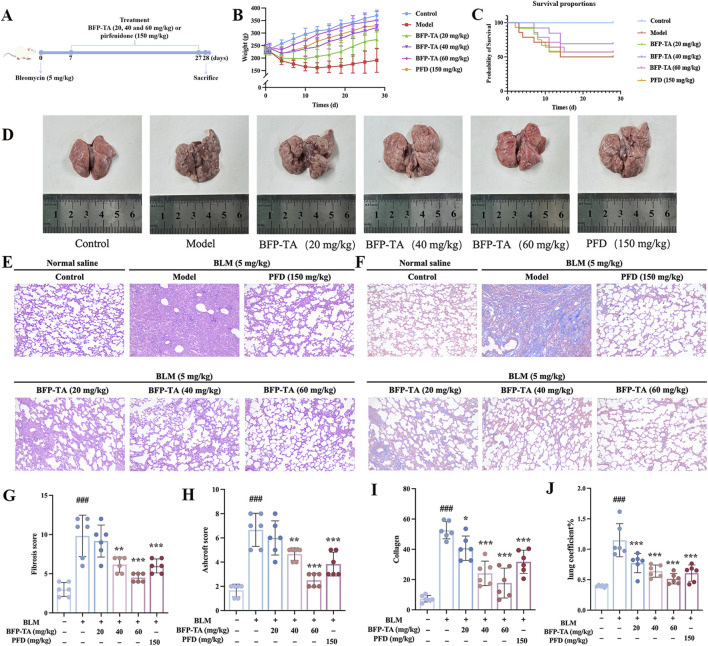
Effects of BFP-TA on lung injury in rats.**(A)** Animal modeling flow chart, **(B)** Body weight change, **(C)** change in survival rate, **(D)** Lung tissue morphology of rats on the 28th day, **(E)** Pathologic HE stained sections of rats, **(F)** Masson stained sections of rats, **(G)** HE scoring of rats, **(H)** Ashcroft score, **(I)** collagen quantification score, **(J)** lung coefficient of rats on the 28th day. ^###^ *P* < 0.001 vs. Control; *P < 0.05, **P < 0.01, ***P < 0.001 vs. Model.

Drawing on the traditional medicinal value of Fritillaria and the enhanced concentration of its bioactive compounds, along with the quest for natural anti-PF remedies, we examined the protective efficacy of BFP-TA against lung injury in PF. One week after the establishment of a rat model of BLM-induced PF, the rats were treated with BFP-TA and PFD for 3 weeks. Acute toxicity experiments were conducted on BFP-TA to evaluate its safety profile and determine the optimal dosage (Supplementary Figure S1). The BLM-induced PF in rats led to symptoms such as weight loss, lethargy, reduced appetite, elevated mortality rate, and increased lung weight-to-body weight ratio. Subsequent administration of BFP-TA and PFD resulted in weight gain, decreased lung weight-to-body weight ratio, and reduced mortality (Figures 3A–C,J). Histological examination demonstrated that BFP-TA treatment mitigated scarring, hemorrhagic lesions, and other pathological manifestations (Figure 3D). Furthermore, histological staining demonstrated that BFP-TA treatment reduced fibroblast proliferation, collagen deposition, and inflammatory cell infiltration, with quantitative analysis revealing substantial differences from the model group (Figures 3E–I). These findings indicate that BFP-TA possesses the ability to ameliorate BLM-induced lung damage.

### 3.4 BFP-TA alleviates the progression of PF by regulating inflammatory factors and ECM

**FIGURE 4:**
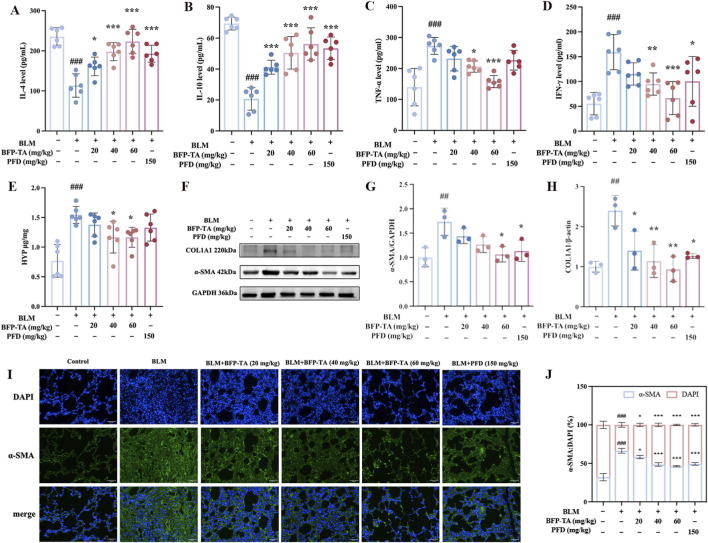
Effects of BFP-TA on lung injury. **(A)** expression of TNF-α in serum, **(B)** expression of ΙL-10 in serum, **(C)** expression of IL-4 in serum, **(D)** expression of IFN-γ in serum, **(E)** expression levels of HYP, **(F)** protein banding diagram, **(G)** protein expression levels of α-SMA, **(H)** protein expression levels of COL1A1, **(I)** immunofluorescence of α-SMA, **(J)** significant difference analysis of α-SMA. ^##^ *P* < 0.01, ^###^ *P* < 0.001 vs. Control; *P < 0.05, **P < 0.01, ***P < 0.001 vs. Model.

The progression of PF in rats comprises two primary stages: the inflammatory stage and the fibrotic stage. The model group exhibited a significant decrease in IL-4 and IL-10 anti-inflammatory cytokine levels, while displaying a notable increase in pro-inflammatory cytokines IL-10 and TNF-α compared to the control group. After BFP-TA treatment, there was a dose-dependent increase in IL-4 and IFN-γ levels, and a dose-dependent decrease in TNF-α and IFN-γ levels (Figures 4A–D). ECM deposition can result in thickening of alveolar septa and alveolar cystic deformation, leading to PF. Upon stimulation of TGF-β signaling, resident fibroblasts are activated, undergo proliferation, and differentiate into myofibroblasts, consequently generating a substantial amount of ECM. Therefore, we assessed the expression of ECM and myofibroblast markers. The findings indicated an improvement in the abnormalities of the primary ECM components, COL1A1, key collagen components, HYP, and the myofibroblast marker α-SMA following BFP-TA treatment (Figures 4E–J).

### 3.5 Proteomics analysis of BFP-TA in alleviating PF in rats

**FIGURE 5:**
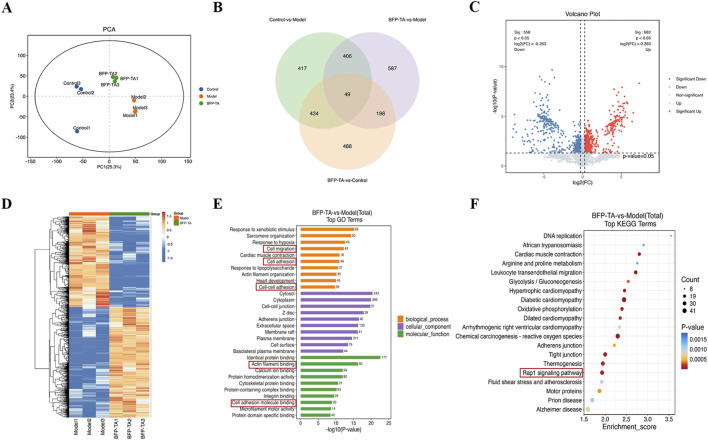
Proteomics analysis. **(A)** PCA diagram, **(B)** Venn diagram, **(C)** volcano diagram of differential proteins in BFP-TA and Model groups, **(D)** Cluster analysis diagram of differential protein expression level, **(E)** GO enrichment analysis Top30 bar graph, **(F)** KEGG enrichment analysis Top20 bubble diagram.

Proteomics analysis was conducted on the BFP-TA group and the model group to explore the mechanism of BFP-TA in alleviating PF. PCA analysis confirmed the validity of the experimental grouping and biological reproducibility, showing high correlation within each group and weak correlation between groups, with data points clustered within each group (Figure 5A). The differential proteins in each group were counted (Figure 5B), and 1,240 differentially expressed proteins were identified between the BFP-TA group and the model group, which highlighted the significant changes in protein abundance between the BFP-TA group and the model group (Figure 5C). It was found that the expression of differential proteins between the BFP-TA group and the Model group was opposite, indicating that BFP-TA played a role in alleviating PF (Figure 5D). Enrichment analysis utilizing Gene Ontology (GO) revealed that BFP-TA predominantly governs biological processes associated with cellular migration and adhesion, encompassing interactions with integrin and binding to actin filaments (Figure 5E). Analysis using the Kyoto Encyclopedia of Genes and Genomes (KEGG) indicates that BFP-TA might mitigate PF by modulating the Rap1 signaling cascade, a pathway linked to cellular adhesion processes (Figure 5F). GESA analysis further demonstrated that the Rap1 signaling pathway is a potential pathway for BFP-TA to resist PF (Supplementary Figure S2).

### 3.6 Transcriptomics and proteomics combined analysis of the molecular mechanism of BFP-TA alleviating PF in rats

**FIGURE 6:**
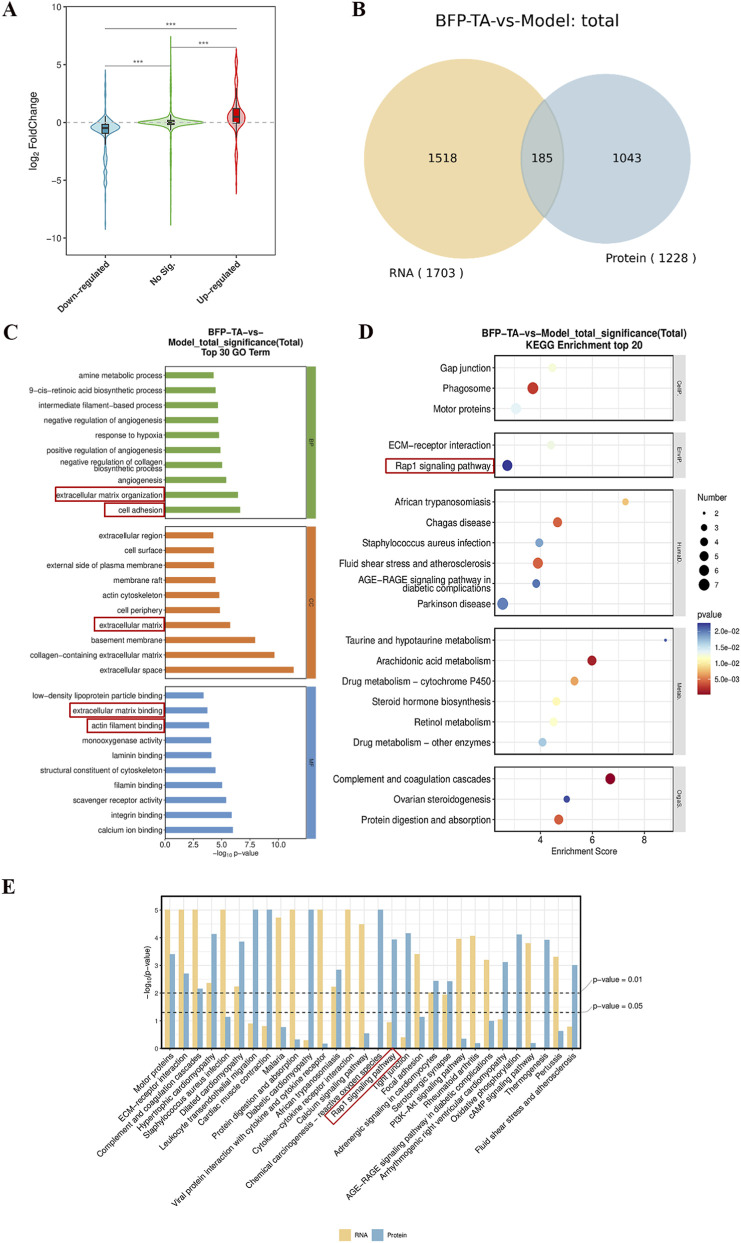
Combined transcriptomics and proteomics analysis. **(A)** difference violin plot, **(B)** difference intersection Venn diagram, **(C)** GO enrichment of intersecting gene enrichment analysis diagrams, **(D)** KEGG enrichment of intersecting gene enrichment analysis diagrams, **(E)** KEGG pathway of pathway enrichment coanalysis.

Through transcriptomic profiling, notable disparities in gene expression patterns were observed when comparing the BFP-TA cohort to the model cohort, with a total of 1,703 genes exhibiting differential expression (Supplementary Figures S3A–C). GO and KEGG enrichment analyses indicated that BFP-TA mitigates PF and is associated with cell adhesion (Supplementary Figures S3D,E). Differential violin plots were created by integrating proteomics and transcriptomics, categorizing genes by their transcriptomic variances in magnitude and direction. These plots depict the distribution of protein expression discrepancies within each group. Significant differences were observed among gene groups, suggesting that variations in protein expression distribution result from biological regulation. Median values were higher in the upregulated transcription group and lower in the downregulated transcription group, suggesting that transcription levels primarily impact protein expression, particularly enriching functions related to signal pathway activation (Figure 6A). A total of 185 genes with differential expression were detected between the BFP-TA group and the model group (Figure 6B). GO and KEGG enrichment analyses of these differentially expressed genes demonstrated that BFP-TA alleviated PF and was linked to biological processes related to cell migration and adhesion, as well as the Rap1 signaling pathway (Figures 6C,D). Subsequently, a joint analysis of the KEGG total enrichment results was conducted, selecting pathways with significant enrichment (*P* < 0.05) for intersection analysis, which revealed a notable distribution of the Rap1 signaling pathway (Figure 6E). Through comparing the differentially expressed genes and proteins within the Rap1 signaling pathway, we identified Rap1 as a differentially expressed gene in this pathway, suggesting that Rap1 could be a target of BFP-TA for mitigating PF. Our statistical analysis focused on differentially expressed genes associated with cell migration and adhesion in the Rap1 signaling pathway (Supplementary Table S5), leading us to focus on the cell migration and adhesion processes modulated by the Vav2, Rac1, and PAK1 genes.

### 3.7 BFP-TA may exert Anti-PF effects via regulation of the Rap1/ERK signaling pathway, cell migration and cell adhesion

**FIGURE 7:**
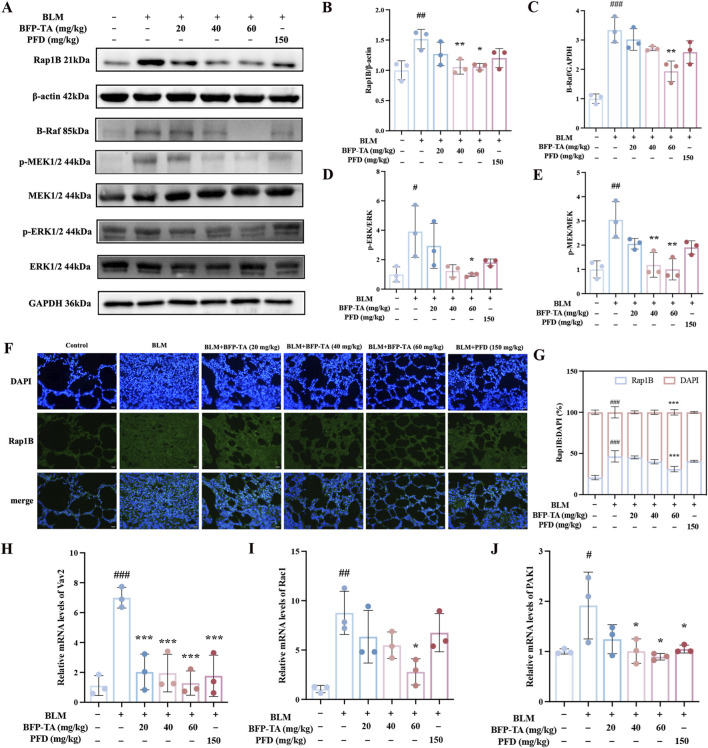
Effects of BFP-TA on anti-PF via Rap1/REK signaling pathway, cell migration and cell adhesion. **(A)** protein banding diagram, **(B)** Rap1B statistic diagram, **(C)** B-Raf statistic diagram, **(D)** p-MEK/MEK statistic diagram, **(E)** p-ERK/ERK statistic diagram, **(F)** immunofluorescence of Rap1B, **(G)** significant difference analysis of Rap1B, **(H)** Vav2 statistic diagram, **(I)** Rac1 statistic diagram, **(J)** PAK1 statistic diagram. ^#^ *P* < 0.05, ^##^ *P* < 0.01, ^###^ *P* < 0.001 vs. Control; *P < 0.05, **P < 0.01, ***P < 0.001 vs. Model.

Sustaining ERK activation in pulmonary fibroblasts is crucial for fibrosis development. Rap1B sustains ERK activation, which promotes fibroblast proliferation and α-SMA expression (Shah et al., 2019; Wei et al., 2023). Furthermore, the activation of Rap1B also influences integrin binding, thereby enhancing cell adhesion to the ECM (Liao et al., 2021). In order to explore whether BFP-TA may inhibit PF by regulating Rap1/ERK pathway, we assessed the levels of pivotal proteins in this cascade. Upon BFP-TA treatment, the levels of Rap1B, B-Raf, p-MEK/MEK, and p-ERK/ERK proteins decreased notably in a dose-dependent fashion (Figures 7A–E). Immunofluorescence analysis of the differentially expressed protein Rap1B revealed a substantial decrease in its expression level post-drug administration (Figures 7F,G). In the context of cell adhesion and migration, the levels of RNA expression for Vav2, Rac1, and PAK1 decreased notably following BFP-TA treatment, showing a dose-dependent decline (Figures 7H–J).

### 3.8 BFP-TA and imperialine may inhibit TGF-β1-induced A549 cells via regulation of the Rap1/ERK signaling pathway

**FIGURE 8:**
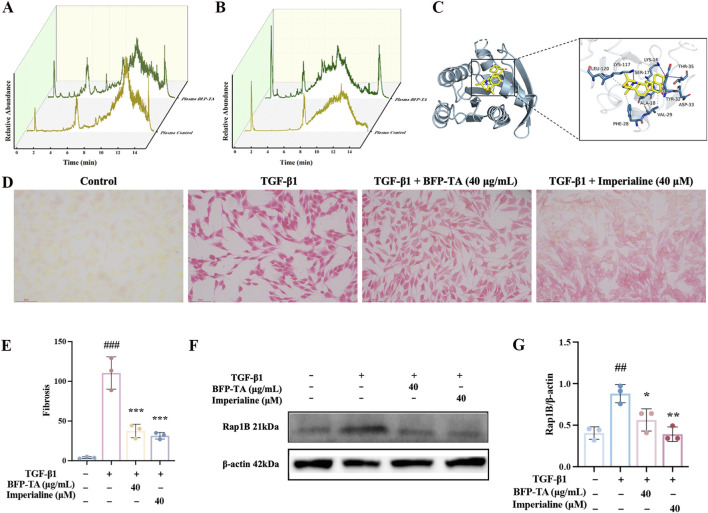
Effects of BFP-TA and imperialine on TGF-β1-induced A549 cells model *in vitro* via Rap1/ERK signaling pathway. **(A)** 3D positive ion comparison, **(B)** 3D negative ion comparison, **(C)** molecular docking, **(D)** sirius red staining diagram, **(E)** statistical analysis bar diagram, **(F)** protein banding diagram, **(G)** effects of imperialine and BFP-TA on TGF-β1-induced Rap1B protein levels in A549 cells, ^##^ *P* < 0.01, ^###^ *P* < 0.001 vs. Control; *P < 0.05,**P < 0.01, ***P < 0.001 vs. Model.

To investigate the primary active components of BFP-TA that are effective against PF, this study analyzed the blood-entering components of BFP-TA (Figures 8A,B). To identify the compounds with the high blood-entering content, detection limits were established and compared with a standard database, resulting in the identification of 13 classes and 73 compounds (Supplementary Figure S4; Supplementary Table S6). Following a comparison with a self-constructed database of *Fritillaria*, imperialine was ultimately selected for further investigation. In order to evaluate the potential interaction between imperialine and the Rap1B, molecular docking analyses were carried out. The computed binding energy of −8.4 kcal/mol for the interaction between the small molecule and the protein suggests a robust binding affinity between imperialine and Rap1B (Figure 8C). Treatment with imperialine and BFP-TA was shown to restore normal cell morphology and reduce red staining in A549 cells, as evidenced by Sirius red staining results, suggesting that both imperialine and BFP-TA can mitigate fibrosis in A549 cells (Figures 8D,E). Imperialine and BFP-TA notably reduced Rap1B protein expression in TGF-β1-induced A549 cells, which proved that they may play an anti-TGF-β1-induced PF *in vitro* by regulating Rap1 signaling pathway (Figures 8F,G).

## 4 Disscussion

The primary characteristics of PF pathogenesis involve disrupted alveolar epithelium repair following repeated microinjury, aberrant fibroblast activation, and overabundant ECM accumulation (Carmona-Pírez et al., 2025). The injury leads to alveolar epithelial apoptosis and abnormal activation of the epithelium, which in turn produces a variety of growth factors and chemokines, induces the proliferation of intrinsic fibroblasts, and ultimately leads to excessive deposition of ECM (Xu et al., 2022). The transformation of fibroblasts into myofibroblasts is the downstream result of epithelial injury and plays a role in the progression of PF (Lu et al., 2026). These myofibroblasts continue to deposit altered ECM, which in turn leads to abnormal epithelial repair and re-epithelialization (Apostolo et al., 2023). Therefore, understanding the mechanisms of alveolar epithelial injury, repair and fibroblast-to-myofibroblast transformation is crucial for developing novel PF therapies.

**FIGURE 9:**
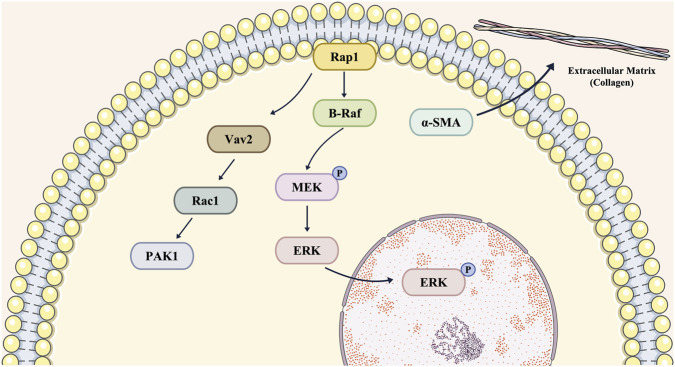
Regulation of BFP-TA on Rap1/ERK signaling pathway, cell migration and cell adhesion process.

In the studies of transcriptomic and proteomic research, the Rap1 signaling cascade has been found as a differential pathway through which BFP-TA exerts its effects against PF. Rap1, a small GTP-binding protein within the Ras superfamily, is crucial for intracellular signaling. It modulates a range of physiological and pathological events by regulating cellular adhesion, motility, proliferation, and differentiation (Branham et al., 2009). Hence, the potential mechanism by which the Rap1 signaling pathway contributes to the pathogenesis of PF might involve the modulation of cellular processes such as adhesion, migration, proliferation, and differentiation. Among the isoforms of Rap1, Rap1B is particularly significant in regulating cellular adhesion and motility (Bos, 2005). Rap1 is upstream regulator of B-Raf. Rap1B activates B-Raf, which in turn activates MEK kinase, which further phosphorylates ERK, forming the MEK-ERK signaling axis. ERK translocates to the nucleus, phosphorylating transcription factors. ERK activation induces ECM protein expression, including collagen and fibronectin (Figure 9) (Wang et al., 2024a; Wang et al., 2024b; Shah et al., 2019). SERK signaling pathway plays a core regulatory role in the process of pulmonary fibrosis. On the one hand, it affects the damage of alveolar epithelial cells, and on the other hand, it promotes the transformation of fibroblasts into myofibroblasts. Studies have shown that increased phosphorylation levels of ERK can induce apoptosis of alveolar epithelial cells, and inhibition of ERK signaling pathway can reduce epithelial cell damage and maintain alveolar epithelial integrity (Sang and Qiao, 2024). In addition, inhibition of ERK phosphorylation can significantly reduce the expression of α-SMA and collagen deposition, thereby reducing tissue fibrosis (Zheng R. et al., 2025). Experiments *in vivo* demonstrated that BFP-TA could decrease the protein levels of Rap1B, B-Raf, MEK1/2, and ERK1/2, indicating a potential role of BFP-TA in slowing PF advancement through modulation of the Rap1/ERK pathway.

Cell migration involves cells moving in response to migratory signals, while cell adhesion is the specific binding process between cells or between cells and the ECM facilitated by adhesion proteins. Integrins link the ECM to the intracellular actin skeleton via α/β heterodimers to form adhesion spots. During cell migration, adhesion plaques assemble at the leading edge of the cell to provide traction and dissociate at the trailing edge to release the cell tail. Rap1 is able to stimulate integrin-mediated adhesion and migration in a wide range of mammalian cell types. Rap1 activation could induce abnormal fibroblast migration and adhesion, enhance adhesion plaque maturation, and potentially expedite PF advancement (Wittchen et al., 2011; Rho et al., 2019). This mechanism likely includes Rap1 binding to Vav2 via its DH-PH domain and translocation to the cell’s forefront. Subsequent Rac1 activation downstream converts it to an active GTP form and interacts with its downstream target PAK1 (Novikov et al., 2021; Byrne et al., 2016), inducing its open active conformation. Hardening of the ECM or an oncogenic mutation results in the sustained activation of the pathway, which in turn drives the deposition of fibrotic ECM (Figure 9) (Mao et al., 2023; Pinilla-Macuaet al., 2025; Qian et al., 2012). The study revealed that BFP-TA enhanced the mRNA levels of Vav2, Rac1, and PAK1. Transcriptomic analysis indicated that these genes were differentially expressed, implying that BFP-TA might mitigate PF advancement by modulating cell adhesion and migration.

Active herbal ingredients, such as flavonoids, terpenoids, and alkaloids, exhibit significant potential in PF treatment. The primary active ingredients in BFP-TA are alkaloids (Liu et al., 2023). Qualitative and quantitative analysis of the chemical composition of BFP-TA revealed that imperialine was the more abundant alkaloid. In addition, imperialine is the index component of the quality control of *Fritillariae Pallidiflorae* Bulbus stipulated in the 2025 edition of the Chinese Pharmacopoeia. Imperialine is not only a blood component detected in this study, but also a key active compound isolated, identified and continuously studied from *Fritillaria* in our previous work. Recent research indicates that imperialine exhibits a range of protective effects targeting multiple aspects in the management of pulmonary diseases, notably including anti-inflammatory properties, antioxidant capabilities, the ability to alleviate pulmonary edema, and anti-tumor effects (Lin et al., 2019; Xu et al., 2016). Moreover, multiple research investigations have demonstrated that, when compared to alternative alkaloids present in *Fritillaria*, imperialine possesses superior anti-inflammatory capabilities (Liu C. S. et al., 2020; Liu S. et al., 2020). However, the precise impact and mechanism by which imperialine operates as an anti-PF agent remain unclear. Utilizing A549 cells, we constructed an *in vitro* model of TGF-β1-induced PF. Through this model, it was observed that imperialine demonstrates anti-fibrotic effects by reducing the expression levels of Rap1B, a protein within the Rap1/ERK signaling pathway.

In conclusion, by regulating the Rap1/ERK signaling pathway and the biological processes of cell adhesion and migration, BFP-TA may lessen BLM-induced PF in rats. The results of the present study provide a strong scientific basis for conducting future clinical trials of BFP-TA for the treatment of PF. In addition, we used a combined multi-omics analysis approach to explore whether BFP-TA might alleviate PF by regulating the biological processes of cell adhesion and cell migration in the Rap1/ERK signaling pathway, which provides ideas for future therapeutic studies of PF.

## 5 Conclusion

In decision, this study used transcriptomics and proteomics to clarify the mechanism of action of BFP-TA against PF, providing the theoretical and material foundation for examining BFP activity and establishing the framework for a new anti-PF drug.

## Data Availability

The original contributions presented in the study are included in the article/Supplementary Material, further inquiries can be directed to the corresponding author.
