# Supplementary material for: The effect and mechanism of total alkaloids of Fritillariae Pallidiflorae Bulbus in alleviating pulmonary fibrosis
Source: Front Pharmacol. 2026 Jul 8;17:1849543. doi: 10.3389/fphar.2026.1849543 (PMC13388258; doi:10.3389/fphar.2026.1849543)
Supplement: Supplementary file 1 [file Supplementaryfile1.docx]

Supplementary Material

# Methods

## Extraction and purification methods of BFP-TA

Take 9.5 kg BFP medicinal materials, crush and sieve, 70% ethanol was added at a solid-liquid ratio of 1:10, refluxed twice, and filtered and dried to obtain the extract of BFP. The pretreated cation exchange resin was loaded into the chromatography column at a diameter-to-height ratio of 1:3. The extract of BFP was dissolved in 2% hydrochloric acid, eluted with 8 BV RO water, and then eluted with 10 BV 5% sodium chloride 60% ethanol solution. The filtrate was concentrated to dryness, dissolved in anhydrous ethanol at a ratio of 1:4, and the pH was adjusted to neutral with high-concentration NaOH. The crude total alkaloids of BFP were obtained by heating and reflux concentration. The pretreated macroporous adsorption resin was then loaded into the chromatography column at a diameter-to-height ratio of 1:3, the crude product was dissolved with 2% hydrochloric acid, the pH was adjusted to 7, 7 BV RO water and 6 BV 20% ethanol solution were used to remove impurities and elute, and then 10 BV 90% ethanol solution and 4 BV anhydrous ethanol solution were used to elute, and the filtrate was concentrated to dryness to obtain 15.8g BFP-TA.

## Qualitative and quantitative analysis of BFP-TA

HPLC-Q-TOF-MS/MS technology was used to qualitatively analyze the chemical components in BFP-TA. For chromatographic conditions: The Agilent ZORBAX SB-C18 Rapid Resolution HD column was selected, with specifications of 100 × 2.1 mm and a particle size of 1.8 μm. Mobile phase composition: Phase A was composed of a 0.1% formic acid aqueous solution, and phase B was acetonitrile. The gradient elution program was set as follows: 0-0.5 min, maintaining the proportion of phase B at 2%; 0.5-10 min, the proportion of phase B increased linearly from 2% to 95%; 10-12 min, maintaining the proportion of phase B at 95%; 12-12.1 min, the proportion of phase B dropped rapidly to 2%; 12.1-15 min, continue to maintain the proportion of phase B at 2%. The mobile phase flow rate was set to 0.3 mL/min, and the injection volume was set to 2 μL. For mass spectrometry conditions: the scanning time of the primary mass spectrometry and the secondary mass spectrometry was 0.662 s, the entire acquisition process lasted 15 min, and the mass range covered was between 50 and 1200 Da. The flow rate of the nebulizer gas was set to 50 psi, the flow rate of the desolvation gas was also 50 psi, the flow rate of the curtain gas was 35 psi, and the temperature of the desolvation gas was set to 500 ℃. The positive ion mode was used for spraying, the voltage was set to 5500 V, and the declustering voltage was 80 V. In mass spectrometry analysis, the primary collision energy was set to 10 V, and the secondary collision energy was set to 50 V.

The 9 alkaloid components in BFP-TA were preliminarily analyzed by HPLC-ELSD technology. The Ultimate XB-C18 column (250 mm × 4.6 mm, 5 μm) was used. According to the method previously reported by our research group, acetonitrile (A) and 0.03% diethylamine (B) were used for gradient elution. The elution gradient was 0-10 min, 30% A; 10-35 min, 30%→60% A; 35-45 min, 60% A; 45-65 min, 60%-90% A; 65-75 min, 90% A; 75-80 min, 90%→30% A; 80-90min, 30% A. The flow rate was 1 mL·min-1, the injection volume was 20 μL; the SEDERE SEDEX 90 evaporative light scattering detector was used, the vaporization temperature was 40 ℃, and the air pressure was 3.5 bar.

LC-MS/MS technology was used to quantitatively analyze the nine alkaloid components in BFP-TA. Chromatographic conditions: Agilent ZORBAX SB-C18 (1.8 μm, 2.10 mm × 100 mm I.D.) column, column temperature 40 ℃. Mobile phase: 0.1% formic acid aqueous solution (A) and acetonitrile (B) gradient elution: 0.0-0.5 min, 15% B; 0.5-3.5 min, 15%→45% B; 3.5-4.5 min, 45%→50% B; 4.5-6.0 min, 50%→95% B; 6.0-8.0 min, 95% B; 8.0-8.1 min, 95%→15% B; 8.1-11.0 min, 15% B. Flow rate: 0.30 mL∙min-1, injection volume 1 μL. For mass spectrometry conditions: ESI source; positive ion monitoring mode, spray voltage set to 5500 V; ion source temperature is 450 ℃. Nebulizer gas (Gas1) 50.0 psi, heating gas (Gas2) 50.0 psi, curtain gas (CUR) 35.0 psi, and nitrogen gas flow throughout the process. Scanning mode uses multiple reaction monitoring; collision gas (CAD) pressure is 9.0; and Q1 and Q3 resolution are both UNIT.

## Transcriptomics

The transcriptome and proteomics (DIA) sequencing and analysis were performed by OE Biotech Co., Ltd. (Shanghai, China). Total RNA was extracted following the operational guidelines of Trizol reagent. The purity of the RNA samples was assessed using a NanoDrop 2000 spectrophotometer, while their concentration was measured; the integrity of the RNA samples was evaluated with an Agilent 2100 bioanalyzer. The construction of the transcriptome sequencing library was completed in accordance with the VAHTS Universal V5 RNA-seq library construction kit manual. The constructed library was sequenced on the Illumina Novaseq 6000 platform, yielding 150 bp paired-end sequencing data. Quality control filtering of the raw sequencing data in fastq format was conducted using the fastp tool. The sequencing data were aligned to the reference genome with HISAT2, and gene expression quantification was performed based on the FPKM method. In the transcriptome data analysis, HTSeq-count software was employed for gene expression quantification, comparing the number of sequencing fragments in specific genomic regions, and systematically calculating the expression level of each gene. Principal component analysis (PCA) was performed on gene expression profile data using the R programming language (v3.2.0) to define sample features. Differential expression analysis utilized DESeq2 software to detect differentially expressed genes (DEGs). Subsequent hierarchical clustering analysis was carried out with R version 3.2.0. Radar charts were generated with the ggradar R package to illustrate expression trends of the top 30 genes with the most significant changes, both up-regulated and down-regulated. Functional annotation analysis of the DEGs was conducted following statistical principles using the hypergeometric distribution algorithm. Integration of the GO and KEGG pathway databases enabled systematic assessment of functional enrichment across biological processes, molecular functions, and cellular components. The outcomes of the functional enrichment analysis were visualized using the R programming language (v3.2.0) through the creation of bar charts, chordal network diagrams, and circular enrichment diagrams.

## Acute toxicity experimental animals and experimental design

Preliminary experiment: 1 SPF Kunming mouse (female) was fasted for 12 hours but not water deprived. The mouse was gavaged with 2000 mg/kg of BFP-TA suspension, and the administration volume was 0.1-0.8 mL/10 g body weight. The experimental animals were closely observed for 48 hours, and the behavioral characteristics, mental state, food and water intake, and the time of death and related symptoms after administration were observed and recorded. If the experimental animal dies within 48 hours, a formal test should be conducted. If the experimental animals survive within 48 hours of the observation period, another 4 experimental animals should be selected and given the same dose of BFP-TA solution. If 3 or more of the 5 animals die, the formal test process should be initiated; on the contrary, if the number of surviving experimental animals exceeds 3, it means that this test can be terminated, indicating that the LD50 of BFP-TA is > 2000 mg/kg.

Formal experiment: 10 SPF Kunming mice (female) were adaptively raised in the standardized animal room of West China School of Pharmacy, Sichuan University for 3-5 days. According to the results of the preliminary test, the initial dose was selected as 175.00 mg/kg, and the dose level factor was 3.2. The acute toxicity test was carried out by single oral administration. After administration, the dose was closely observed for 48 hours. The next level of administration was determined according to the test results. If the experimental animal died, the dose was reduced by 1.5 times, and if the experimental animal survived, the dose was increased by 1.5 times. The body weight of the experimental mice was measured every 24 hours, and the observation was continued for 14 days, and their appearance characteristics and behavioral changes were recorded in detail. After 14 days, the surviving animals were killed, the death weight was recorded, and the autopsy was carried out in time. During the autopsy, the morphological changes of important organs (heart, spleen, liver, kidney, lung, and stomach) were observed with the naked eye, and samples of the above major organs were quickly removed and washed in pre-cooled physiological saline. After wiping off the floating water, the weight of each organ was weighed and fixed in 4% paraformaldehyde solution for 48 hours. The specimens were then subjected to histopathological examination.

# Supplementary Figures
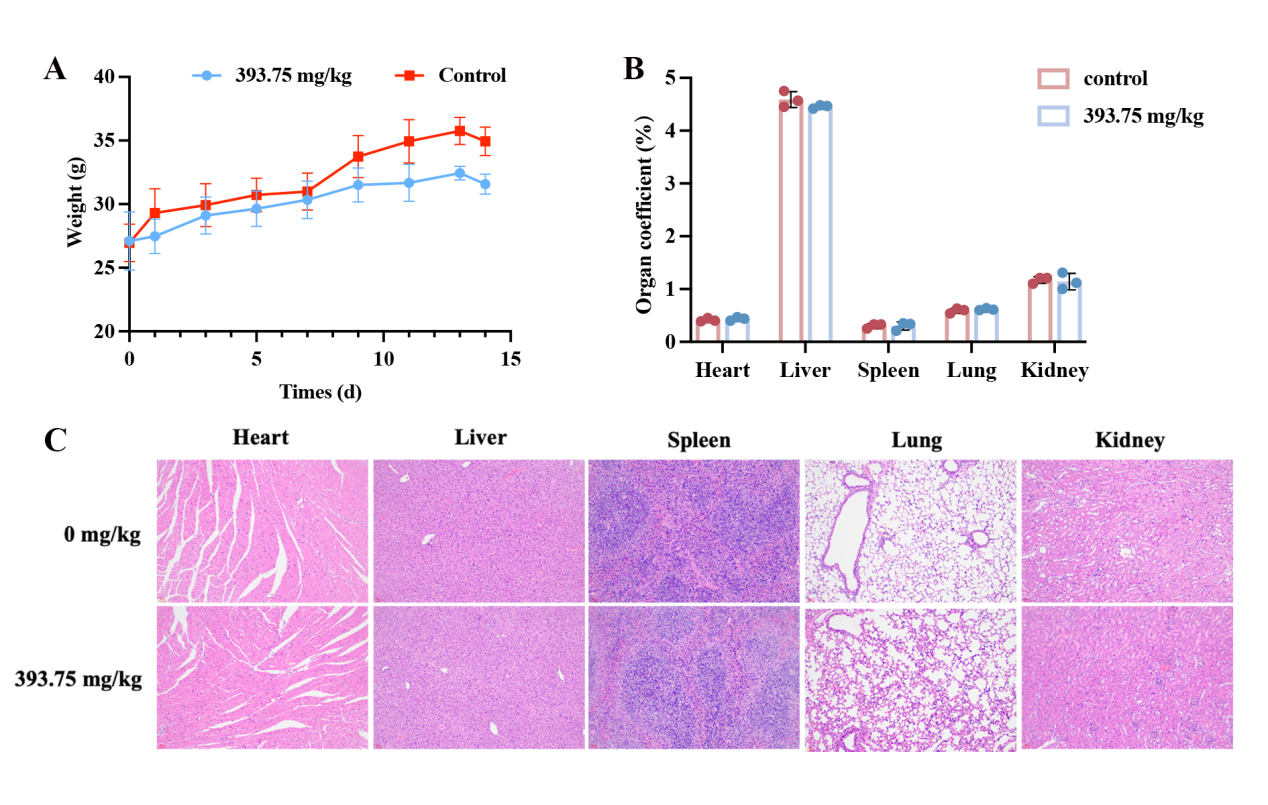


## Supplementary Figure 1. Acute toxicity experiment for the ‘up and down method’. (A) Body weight changes in surviving KM mice, (B) Organ coefficients of mice in the 393.75 mg/kg dose group and blank group, (C) HE-stained sections of organs of mice surviving acute toxicity experiments with BFP-TA: heart, liver, spleen, lungs, kidneys (10×).


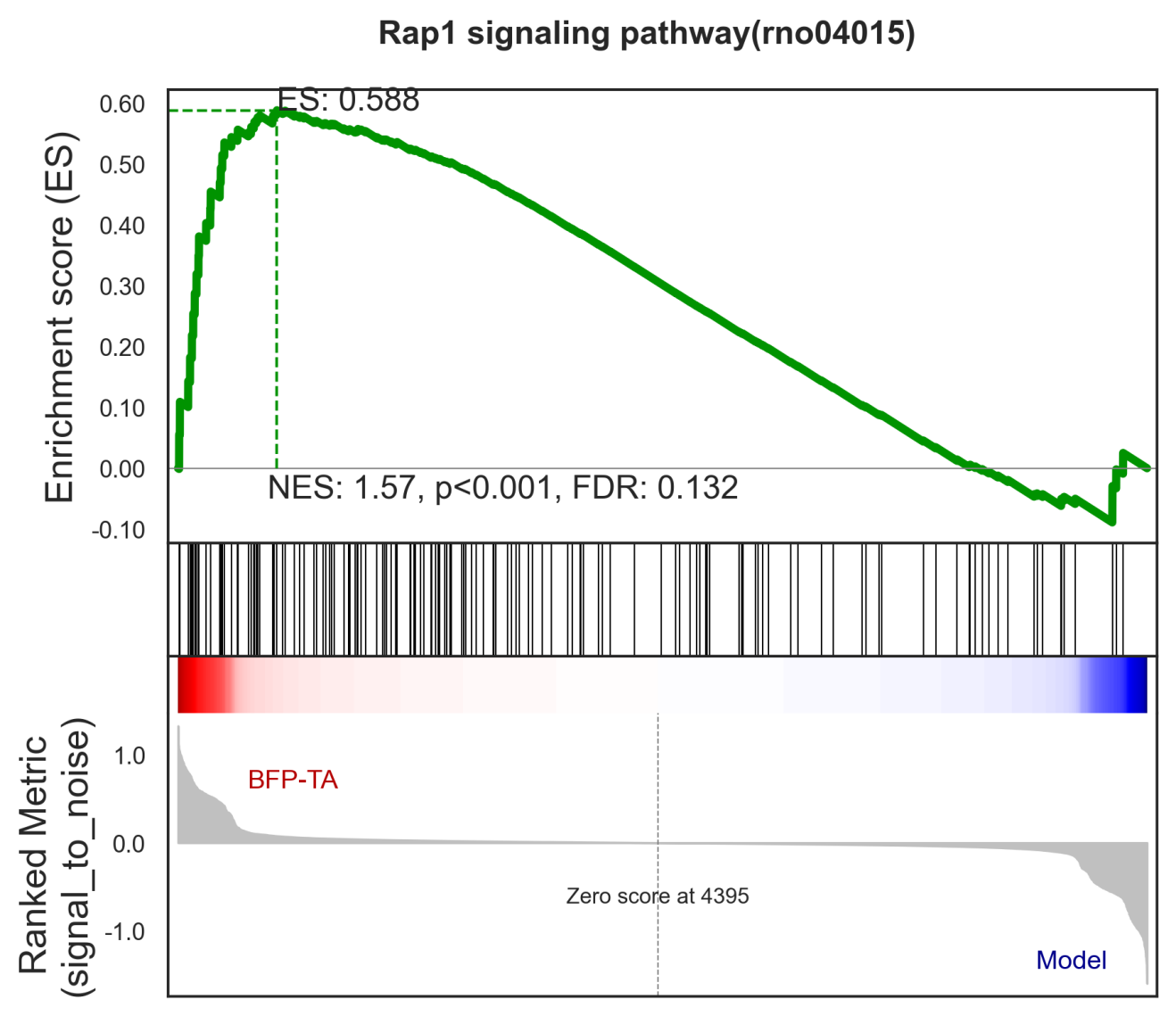


**Supplementary Figure 2.** GSEA KEGG enrichment analysis diagram.

##
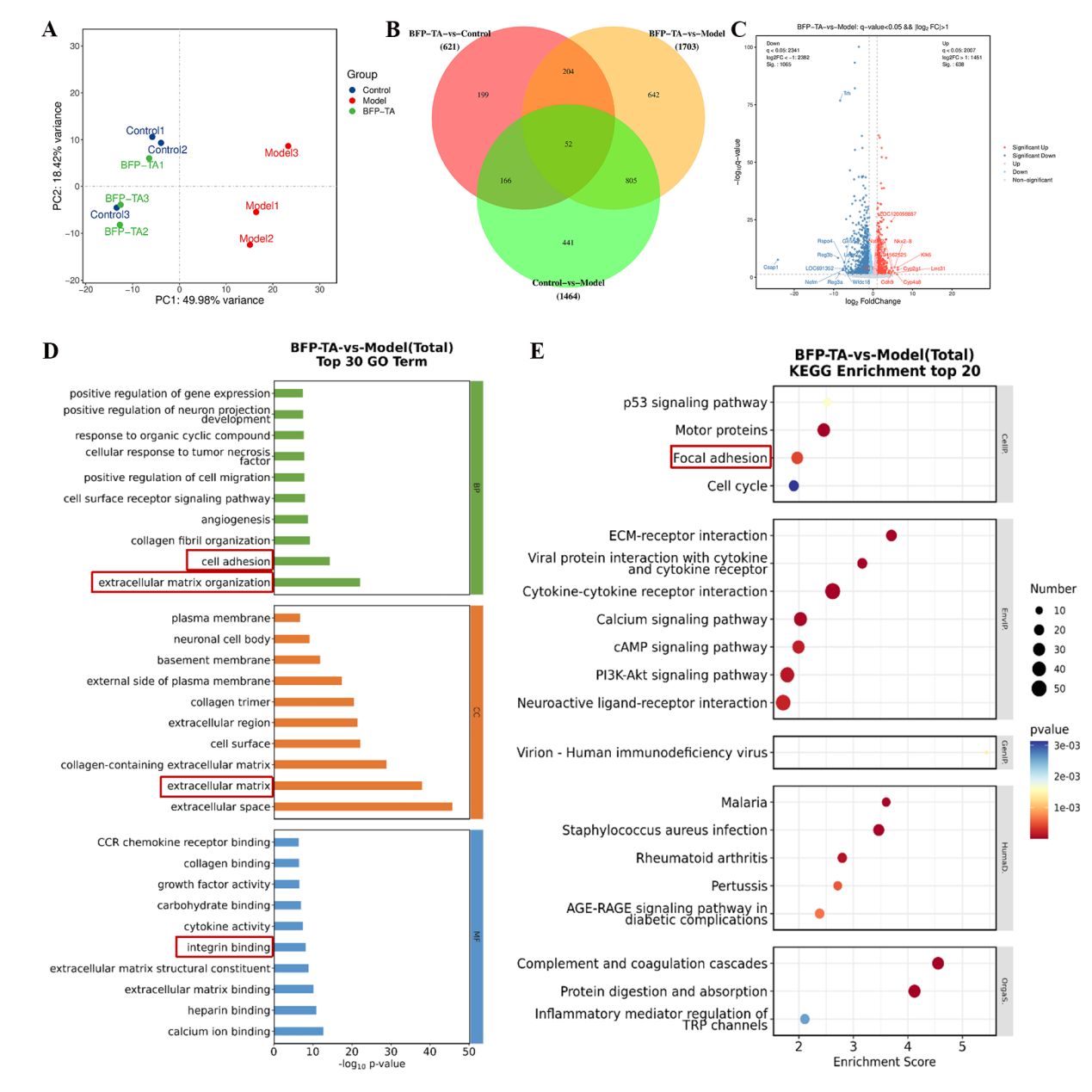


## Supplementary Figure 3. Transcriptomic analysis. (A) PCA diagram, (B) statistical diagram of the number of differentially expressed genes, (C) volcano diagram of differential genes in BFP-TA and Model groups, (D) GO enrichment analysis Top30 bar graph, (E) KEGG enrichment analysis Top20 bubble diagram.


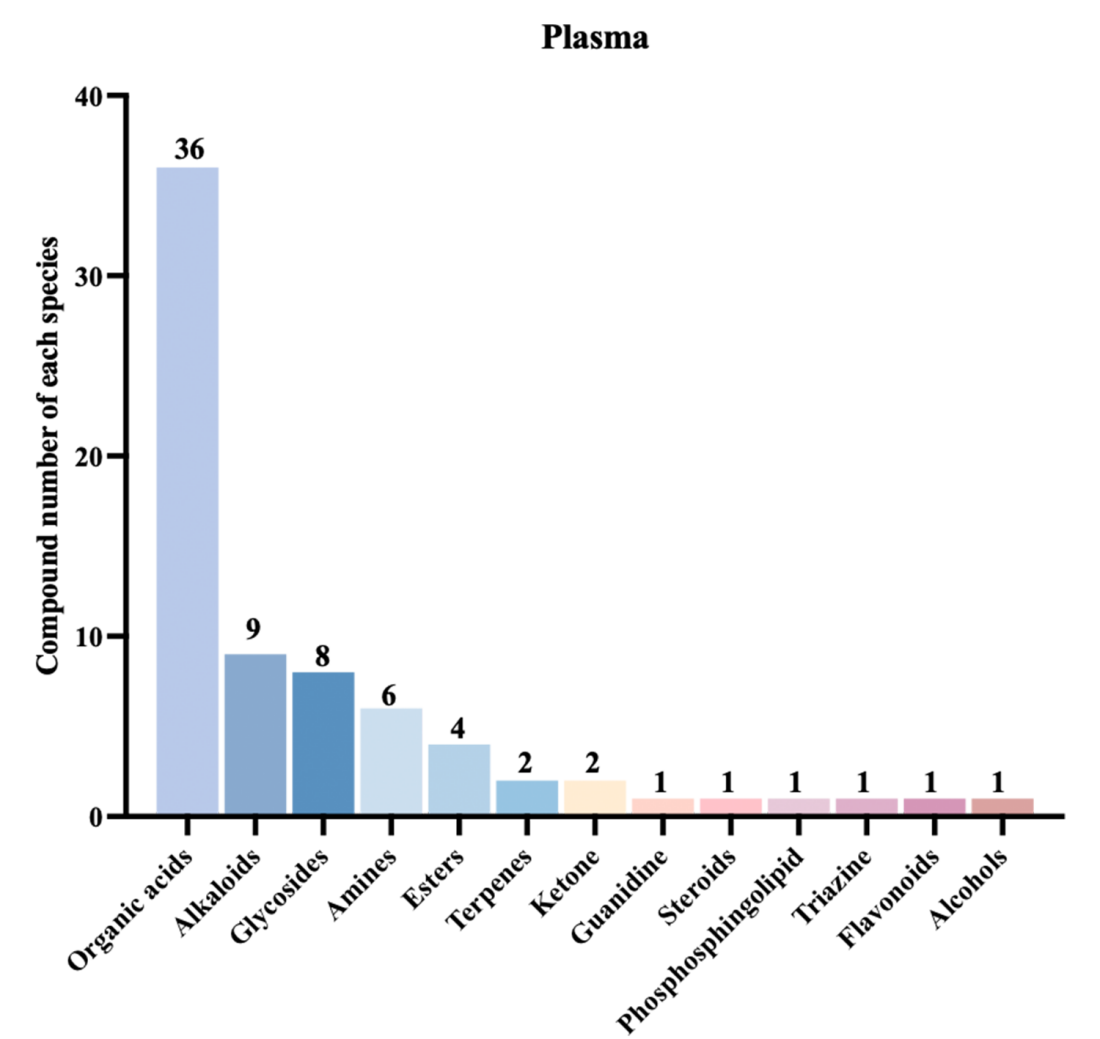


## Supplementary Figure 4. Types and amounts of components contained in rat plasma.
