# Supplementary material for: The effect and mechanism of total alkaloids of Fritillariae Pallidiflorae Bulbus in alleviating pulmonary fibrosis
Source: Front Pharmacol. 2026 Jul 8;17:1849543. doi: 10.3389/fphar.2026.1849543 (PMC13388258; doi:10.3389/fphar.2026.1849543)
Supplement: Supplementary file 2 [file Supplementaryfile2.docx]

**Table. S1** Gene names and polymerase chain reaction (PCR) primer sequences.

| Gene | Forward Primer (5’→3’) | Reverse Primer (5’→3’) |
| --- | --- | --- |
| Vav2 | GGAGGCAGGCAGGACCAGAG | AGGGAGGCAGCCAGGGAAATC |
| Rac1 | AGCACTCACACAGCGAGGACTC | AAAGGCTCCAGGGACCAAGACC |
| PAK1 | CCACTCCGCCAGATGCTTTGAC | GGATCGCCCACACTCACTATGC |

**Table. S2** HPLC-Q-TOF-MS/MS identification of BFP-TA.

| **No.** | **Compound Name** | **CAS** | **Molecular Formula** | **Classification** |
| --- | --- | --- | --- | --- |
| **1** | Arginine | 74-79-3 | C_6_H_14_N_4_O_2_ | Organic acids |
| **2** | Choline | 62-49-7 | C_5_H_14_NO^+^ | Alkaloids |
| **3** | TREHALOSE | 99-20-7 | C_12_H_22_O_11_ | Glycosides |
| **4** | ADENINE | 73-24-5 | C_5_H_5_N_5_ | Purine |
| **5** | Adenosine | 58-61-7 | C_10_H_13_N_5_O_4_ | Glycosides |
| **6** | Ecgonine-methyl-ester (EME) | 7143-09-1 | C_10_H_17_NO_3_ | Esters |
| **7** | 4-(Dimethylamino)pyridine | 1122-58-3 | C_7_H_10_N_2_ | Alkaloids |
| **8** | 1-Methyladenine | 5142-22-3 | C_6_H_7_N_5_ | Purine |
| **9** | Guanine | 73-40-5 | C_5_H_5_N_5_O | Purine |
| **10** | Guanosine | 118-00-3 | C_10_H_13_N_5_O_5_ | Purine nucleosides |
| **11** | cordycepin | 73-03-0 | C_10_H_13_N_5_O_3_ | Glycosides |
| **12** | N,N-dimethyl-7H-purin-6-amine | 938-55-6 | C_7_H_9_N_5_ | Purine |
| **13** | 2-O-Methyladenosine | 42173-86-4 | C_11_H_15_N_5_O_4_ | Purine nucleosides |
| **14** | Phenylalanine | 63-91-2 | C_9_H_11_NO_2_ | Organic acids |
| **15** | Galbelgin | 10569-12-7 | C_22_H_28_O_5_ | Organooxygen Compounds |
| **16** | Indole-3-carbinol | 700-06-1 | C_9_H_9_NO | Alkaloids |
| **17** | N'-(2,4-Dimethylphenyl)-N-methylformamidine | 33089-74-6 | C_10_H_14_N_2_ | Alkaloids |
| **18** | 5'-S-Methylthioadenosine | 2457-80-9 | C_11_H_15_N_5_O_3_S | Purine nucleosides |
| **19** | Tryptophan | 54-12-6 | C_11_H_12_N_2_O_2_ | Organic acids |
| **20** | 2-Naphthylamine | 91-59-8 | C_10_H_9_N | Amines |
| **21** | Maltol | 118-71-8 | C_6_H_6_O_3_ | Ketone |
| **22** | Harmaline | 304-21-2 | C_13_H_14_N_2_O | Alkaloids |
| **23** | (S)-4-((7-acetamido-1,2,3-trimethoxy-9-oxo-5,6,7,9-tetrahydrobenzo[a]heptalen-10-yl)amino)-N-(benzo[d][1,3]dioxol-5-ylmethyl)butanamide | NA | C_33_H_37_N_3_O_8_ | Organic acids |
| **24** | 4-Hydroxyquinoline | 529-37-3 | C_9_H_7_NO | Alkaloids |
| **25** | triacanthine | 10091-84-6 | C_10_H_13_N_5_ | Purine |
| **26** | 1-Benzylimidazole | 4238-71-5 | C_10_H_10_N_2_ | Alkaloids |
| **27** | Pseudojervine | 36069-05-3 | C_33_H_49_NO_8_ | Steroids |
| **28** | (-)-Riboflavin | 83-88-5 | C_17_H_20_N_4_O_6_ | Flavonoids |
| **29** | Norharmane | 244-63-3 | C_11_H_8_N_2_ | Alkaloids |
| **30** | 1-tridecanoyl-2-hydroxy-sn-glycero-3-phosphocholine | 20559-17-5 | C_21_H_44_NO_7_P | Alkaloids |
| **31** | Harmane | 486-84-0 | C_12_H_10_N_2_ | Alkaloids |
| **32** | **Edpetiline** | 32685-93-1 | C_33_H_53_NO_8_ | Alkaloids |
| **33** | Solasonine | 19121-58-5 | C_45_H_73_NO_16_ | Steroids |
| **34** | 1,1,2-Trimethyl-1H-benzo[e]indole | 41532-84-7 | C_15_H_15_N | Alkaloids |
| **35** | Tramadol | 123154-38-1 | C_16_H_25_NO_2_ | Alcohols |
| **36** | Tramadol N-Oxide | 147441-56-3 | C_16_H_25_NO_3_ | Alcohols |
| **37** | (2R,2'R,4a'S,6'R,8a'S)-4,6'-dihydroxy-7-(2-hydroxyethyl)-2',5',5',8a'-tetramethyl-3',4',4a',5',6',7,7',8,8',8a'-decahydro-2'H-spiro[furo[2,3-e]isoindole-2,1'-naphthalen]-6(3H)-one | 149598-71-0 | C_25_H_35_NO_5_ | Alkaloids |
| **38** | **Peiminine** | 18059-10-4 | C_27_H_43_NO_3_ | Alkaloids |
| **39** | beta-solanine | 61877-94-9 | C_39_H_63_NO_11_ | Steroids |
| **40** | Ergocorninine | 564-37-4 | C_31_H_39_N_5_O_5_ | Alkaloids |
| **41** | **Veratrosine** | 475-00-3 | C_33_H_49_NO_7_ | Alkaloids |
| **42** | **Veratramine** | 60-70-8 | C_27_H_39_NO_2_ | Alkaloids |
| **43** | (+)-Huperzine A | 130791-77-4 | C_15_H_18_N_2_O | Alkaloids |
| **44** | ancistrocladine | 32221-59-3 | C_25_H_29_NO_4_ | Alkaloids |
| **45** | **Cyclopamine** | 4449-51-8 | C_27_H_41_NO_2_ | Alkaloids |
| **46** | Salmeterol | 89365-50-4 | C_25_H_37_NO_4_ | Alcohols |
| **47** | Khasianine | 32449-98-2 | C_39_H_63_NO_11_ | Steroids |
| **48** | alpha-Solanine | 20562-02-1 | C_45_H_73_NO_15_ | Steroids |
| **49** | Tomatidine | 77-59-8 | C_27_H_45_NO_2_ | Steroids |
| **50** | Solasodin | 126-17-0 | C_27_H_43_NO_2_ | Steroids |
| **51** | Glabrol | 59870-65-4 | C_25_H_28_O_4_ | Flavonoids |
| **52** | **Peimine** | 23496-41-5 | C_27_H_45_NO_3_ | Alkaloids |
| **53** | O-Acetylsolasodine | 6159-99-5 | C_29_H_45_NO_3_ | Steroids |
| **54** | N-(3-Methoxy)Benzyllinoleamide | 883715-22-8 | C_26_H_41_NO_2_ | Alkaloids |
| **55** | Platycodigenin | 22327-82-8 | C_30_H_48_O_7_ | Terpenes |
| **56** | 11-Ketotestosterone | 564-35-2 | C_19_H_26_O_3_ | Steroids |
| **57** | 1-Palmitoyl-sn-glycero-3-phosphocholine | 17364-16-8 | C_24_H_50_NO_7_P | Alkaloids |
| **58** | N-(3-Methoxybenzyl)oleamide | 883715-21-7 | C_26_H_43_NO_2_ | Alkaloids |
| **59** | Karacoline | 39089-30-0 | C_22_H_35_NO_4_ | Alkaloids |
| **60** | **Korseveriline** | 1260207-37-1 | C_27_H_45_NO_3_ | Alkaloids |
| **61** | Citrinin | 518-75-2 | C_13_H_14_O_5_ | Organic acids |
| **62** | 4-Methylphthalic anhydride | 19438-61-0 | C_9_H_6_O_3_ | Anhydrides |
| **63** | Dixyrazine | 2470-73-7 | C_24_H_33_N_3_O_2_S | Phenothiazides |
| **64** | Neoruscogenin | 17676-33-4 | C_27_H_40_O_4_ | Terpenes |
| **65** | Gymnemagenin | 22467-07-8 | C_30_H_50_O_6_ | Terpenes |
| **66** | Trospium | 47608-32-2 | C_25_H_30_NO_3_^+^ | Esters |
| **67** | (9Z,12Z)-N-Benzyloctadeca-9,12-dienamide | 18286-71-0 | C_25_H_39_NO | Amines |
| **68** | Ganoderic acid I | 98665-20-4 | C_30_H_44_O_8_ | Terpenes |
| **69** | Solanidine (not validated) | 1264019-92-2 | C_27_H_43_NO | Alkaloids |
| **70** | Diethyltoluamide | 134-62-3 | C_12_H_17_NO | Alkaloids |
| **71** | Aurantiamide | 58115-31-4 | C_25_H_26_N_2_O_3_ | Alkaloids |
| **72** | Triphenylphosphine oxide | 791-28-6 | C_18_H_15_OP | Phosphine Oxidate |
| **73** | Carbaryl | 63-25-2 | C_12_H_11_NO_2_ | Esters |
| **74** | Secbumeton | 26259-45-0 | C_10_H_19_N_5_O | Alkaloids |
| **75** | 16-Deethylindanomycin | 106803-22-9 | C_29_H_39_NO_4_ | Ketone |
| **76** | 3-(3,4-Dihydro-2H-1,5-benzodioxepin-7-yl)-7-ethoxy-2-methyl-4H-1-benzopyran-4-one | 302939-34-0 | C_21_H_20_O_5_ | Flavonoids |
| **77** | Ganoderic Acid H | 98665-19-1 | C_32_H_44_O_9_ | Terpenes |
| **78** | Phthalic anhydride | 85-44-9 | C_8_H_4_O_3_ | Anhydrides |
| **79** | Fluvastatin | 93957-54-1 | C_24_H_26_FNO_4_ | Organic acids |
| **80** | **Jervine** | 469-59-0 | C_27_H_39_NO_3_ | Alkaloids |
| **81** | Mephedrone | 1189805-46-6 | C_11_H_15_NO | Ketone |
| **82** | Fexofenadine | 83799-24-0 | C_32_H_39_NO_4_ | Organic acids |
| **83** | 2-Aminooctadec-8-ene-1,3,4-triol | 81520-97-0 | C_18_H_37_NO_3_ | Amines |
| **84** | tigogenin | 77-60-1 | C_27_H_44_O_3_ | Terpenes |
| **85** | Ziyuglycoside II | 35286-59-0 | C_35_H_56_O_8_ | Terpenes |
| **86** | 1-(9Z,12Z-octadecadienoyl)-sn-glycero-3-phosphocholine | 22252-07-9 | C_26_H_50_NO_7_P | Alkaloids |
| **87** | Pantothenic acid | 79-83-4 | C_9_H_17_NO_5_ | Organic acids |
| **88** | Isocurcumenol | 24063-71-6 | C_15_H_22_O_2_ | Terpenes |
| **89** | Cafestol | 469-83-0 | C_20_H_28_O_3_ | Organic acids |
| **90** | Panaxcerol B | 171520-42-6 | C_27_H_46_O_9_ | Esters |
| **91** | Corynoxeine | 630-94-4 | C_22_H_26_N_2_O_4_ | Alkaloids |
| **92** | Dibutyl phthalate | 84-74-2 | C_16_H_22_O_4_ | Esters |
| **93** | Acetyl tributyl citrate | 77-90-7 | C_20_H_34_O_8_ | Organic acids |
| **94** | Kirenol | 52659-56-0 | C_20_H_34_O_4_ | Terpenes |
| **95** | Darutigenol | 5940-00-1 | C_20_H_34_O_3_ | Terpenes |
| **96** | Dioctyl Phthalate | 117-84-0 | C_24_H_38_O_4_ | Esters |
| **97** | Phthalic acid | 88-99-3 | C_8_H_6_O_4_ | Organic acids |
| **98** | cyclovirobuxine D | 860-79-7 | C_26_H_46_N_2_O | Terpenes |
| **99** | 2-Stearoyl-sn-glycero-3-phosphocholine | 4421-58-3 | C_26_H_54_NO_7_P | Alkaloids |
| **100** | Lactate | 50-21-5 | C_3_H_6_O_3_ | Alkaloids |
| **101** | URATE | 69-93-2 | C_5_H_4_N_4_O_3_ | Purine |
| **102** | Citrate acid | 77-92-9 | C_6_H_8_O_7_ | Organic acids |
| **103** | m-Hydroxycinnamic acid | 14755-02-3 | C_9_H_8_O_3_ | Organic acids |
| **104** | Tyrosine | 60-18-4 | C_9_H_11_NO_3_ | Organic acids |
| **105** | 3-Hydroxybutyric acid | 300-85-6 | C_4_H_8_O_3_ | Organic acids |
| **106** | ISOLEUCINE | 73-32-5 | C_6_H_13_NO_2_ | Organic acids |
| **107** | L-Tryptophan | 73-22-3 | C_11_H_12_N_2_O_2_ | Organic acids |
| **108** | Priverosaponin B 22-acetate | 144379-37-3 | C_56_H_90_O_25_ | Terpenes |
| **109** | 2-Hydroxy-4-methylpentanoate | 13748-90-8 | C_6_H_12_O_3_ | Organic acids |
| **110** | 5-Hydroxy-3-indoleaceacetate | 54-16-0 | C_10_H_9_NO_3_ | Organic acids |
| **111** | Baicalin | 21967-41-9 | C_21_H_18_O_11_ | Flavonoids |
| **112** | Acetylendicarboxylate | 142-45-0 | C_4_H_2_O_4_ | Organic acids |
| **113** | Taurocholic acid | 81-24-3 | C_26_H_45_NO_7_S | Organic acids |
| **114** | sebacic acid | 111-20-6 | C_10_H_18_O_4_ | Organic acids |
| **115** | Indole-3-propionic acid | 830-96-6 | C_11_H_11_NO_2_ | Organic acids |
| **116** | 9,12,13-Trihydroxy-10-octadecenoic acid | 29907-56-0 | C_18_H_34_O_5_ | Organic acids |
| **117** | GLYCOCHOLATE | 475-31-0 | C_26_H_43_NO_6_ | Organic acids |
| **118** | Cholic acid | 81-25-4 | C_24_H_40_O_5_ | Organic acids |
| **119** | hyocholic acid | 547-75-1 | C_24_H_40_O_5_ | Organic acids |
| **120** | Butylparaben | 94-26-8 | C_11_H_14_O_3_ | Esters |
| **121** | Hyodeoxycholic acid | 83-49-8 | C_24_H_40_O_4_ | Organic acids |
| **122** | 9.11(13)-Eremophiladien-12-oic acid | 69905-01-7 | C_15_H_22_O_2_ | Terpenes |
| **123** | Leukotoxin diol | 189191-41-1 | C_18_H_34_O_4_ | Organic acids |
| **124** | Lauryl sulfate | 151-41-7 | C_12_H_26_O_4_S | Organic acids |
| **125** | Sodium laureth sulfate | 48073-44-5 | C_14_H_30_O_5_S | Organic acids |
| **126** | 12,13-Dihydroxy-9-octadecenoic acid | 263399-35-5 | C_18_H_34_O_4_ | Organic acids |
| **127** | Haloxyfop | 69806-34-4 | C_15_H_11_ClF_3_NO_4_ | Organic acids |
| **128** | 1-alpha-linolenoyl-glycero-3-phosphocholine | 62512-91-8 | C_26_H_48_NO_7_P | Alkaloids |
| **129** | 1-linoleoyl-sn-glycero-3-phosphoethanolamine | 85046-18-0 | C_23_H_44_NO_7_P | Amines |
| **130** | Thymol-beta-D-glucoside | 20772-23-0 | C_16_H_24_O_6_ | Terpenes |
| **131** | 8-hydroxy-5Z,9E,11Z,14Z-eicosatetraenoic acid | 79495-84-4 | C_20_H_32_O_3_ | Organic acids |
| **132** | Octadecanedioic acid | 871-70-5 | C_18_H_34_O_4_ | Organic acids |
| **133** | Canrenone | 976-71-6 | C_22_H_28_O_3_ | Steroids |
| **134** | 2,6-Di-tert-butyl-4-nitrophenol | 728-40-5 | C_14_H_21_NO_3_ | Phenols |
| **135** | Abietic acid | 514-10-3 | C_20_H_30_O_2_ | Terpenes |
| **136** | a-Linolenic acid | 463-40-1 | C_18_H_30_O_2_ | Organic acids |
| **137** | Docosahexaenoic acid | 6217-54-5 | C_22_H_32_O_2_ | Organic acids |
| **138** | 9-Trans-Palmitelaidic acid | 10030-73-6 | C_16_H_30_O_2_ | Organic acids |
| **139** | Arachidonic acid | 506-32-1 | C_20_H_32_O_2_ | Organic acids |
| **140** | (9Z, 12Z)-Octadecadienoate | 60-33-3 | C_18_H_32_O_2_ | Organic acids |
| **141** | Palmitic acid | 57-10-3 | C_16_H_32_O_2_ | Organic acids |
| **142** | Oleic acid | 112-80-1 | C_18_H_34_O_2_ | Organic acids |
| **143** | Cyclohexanecarboxylate | 3198-23-0 | C_7_H_12_O_2_ | Organic acids |
| **144** | 4-((9S)-3,5,14-trihydroxy-10-((E)-((1-hydroxybutan-2-yl)imino)methyl)-13-methylhexadecahydro-1H-cyclopenta[a]phenanthren-17-yl)furan-2(5H)-one | NA | C_27_H_41_NO_6_ | Steroids |
| **145** | 8-Hydroxy-9,10-epoxystearic acid | NA | C_18_H_34_O_4_ | Organic acids |
| **146** | (3S,6S,6aR,7R,7aR,8S,9R,10S,11aR,12R,13S,14R)-1-ethyl-3-(hydroxymethyl)-6,8,10,13-tetramethoxytetradecahydro-1H-3,6a,12-(epiethane[1,1,2]triyl)-7,9-methanonaphtho[2,3-b]azocine-11a,12-diol | NA | C_25_H_41_NO_7_ | Alkaloids |
| **147** | 3-(benzo[d]thiazol-2-yl)-2-methyl-4-oxo-8-(pyrrolidin-1-ium-1-ylmethyl)-4H-chromen-7-olate | NA | C_22_H_20_N_2_O_3_S | Ketone |
| **148** | (S)-7-(2-(2-(hydroxymethyl)pyrrolidin-1-yl)-2-oxoethoxy)-2-methyl-3-phenyl-4H-chromen-4-one | NA | C_23_H_23_NO_5_ | Flavonoids |
| **149** | (5alpha,8xi,9xi,14xi,16xi,17xi)-26-(Acetylamino)furost-20(22)-en-3-yl acetate | NA | C_31_H_49_NO_4_ | Steroids |
| **150** | (Z)-5,8,11-trihydroxyoctadec-9-enoic acid | NA | C_18_H_34_O_5_ | Organic acids |
| **151** | 19S-Methoxytubotaiwine | NA | C_21_H_26_N_2_O_3_ | Alkaloids |
| **152** | 2-(14-Methylpentadecanoylamino)-3-phenylpropanoic acid | NA | C_25_H_41_NO_3_ | Organic acids |
| **153** | (2'R,4R,5'R,6aR,6bS,8aS,8bR,9S,11aS,12aR,12bS)-5',6a,8a,9-tetramethyl-1,3,3',4,4',5,5',6,6a,6b,6',7,8,8a,8b,9,11a,12,12a,12b-icosahydrospiro[naphtho[2',1':4,5]indeno[2,1-b]furan-10,2'-pyran]-4-ol | NA | C_27_H_42_O_3_ | Terpenes |
| **154** | 4-Decan-4-ylbenzenesulonic acid | NA | C_16_H_26_O_3_S | Organic acids |
| **155** | Phosphatidylethanolamine lyso 20:4 | NA | C_25_H_44_NO_7_P | Amines |
| **156** | FA 18:2+2O | NA | C_18_H_32_O_4_ | Organic acids |
| **157** | (Z)-9,12,13-trihydroxyoctadec-15-enoic acid | NA | C_18_H_34_O_5_ | Organic acids |
| **158** | 1-palmitoyl-lysophosphatidylcholine | NA | C_24_H_50_NO_7_P | Alkaloids |
| **159** | sodium (E)-2-((4S)-16-acetoxy-3,11-dihydroxy-4,8,10,14-tetramethyldodecahydro-1H-cyclopenta[a]phenanthren-17(2H,10H,14H)-ylidene)-6-methylhept-5-enoate | NA | C_31_H_47_NaO_6_ | Esters |
| **160** | (1S,3R,6S,6aR,6bR,8S,9S,11R,11aR,12R,12aR,14R)-1-ethyl-6,8,11-trihydroxy-3-methyl-10-methylenetetradecahydro-3,6a,12-(epiethane[1,1,2]triyl)-9,11a-methanoazuleno[2,1-b]azocine 1-oxide | NA | C_22_H_33_NO_4_ | Terpenes |
| **161** | L-Arachidonoylcarnitine | NA | C_27_H_45_NO_4_ | Organic acids |
| **162** | **Ebeinone** | 125409-58-7 | C_27_H_41_NO_2_ | Alkaloids |
| **163** | **Yibeinone B** | 1888337-75-4 | C_27_H_39_NO_3_ | Alkaloids |
| **164** | **Hapepunine** | 68422-01-5 | C_28_H_47_NO_2_ | Alkaloids |
| **165** | 6-[[(3S,6aR,6bS,8aR,14bR)-9-hydroxy-4,4,6a,6b,8a,11,11,14b-octamethyl-1,2,3,4a,5,6,7,8,9,10,12,12a,14,14a-tetradecahydropicen-3-yl]oxy]-5-[4,5-dihydroxy-3-(3,4,5-trihydroxy-6-methyloxan-2-yl)oxyoxan-2-yl]oxy-3,4-dihydroxyoxane-2-carboxylic acid | NA | C_47_H_76_O_16_ | Terpenes |
| **166** | (4Bs,8as,14br)-5,6,7,8,14,14b-hexahydro-7-(2-methyl-2-propenyl)-4,8-methanobenzofuro[2,3-a]pyrido[4,3-b]carbazole-1,8a(9h)-diol | NA | C_26_H_26_N_2_O_3_ | Alkaloids |
| **167** | N-(2-(((1S,9aR)-octahydro-1H-quinolizin-1-yl)methyl)-3-oxo-1,2,3,4-tetrahydrobenzo[4,5]imidazo[1,2-a]pyrazin-8-yl)propionamide | NA | C_23_H_31_N_5_O_2_ | Alkaloids |
| **168** | **Yibeissine** | 143502-51-6 | C_27_H_41_NO_4_ | Alkaloids |
| **169** | **Imperialine N-oxide** | 62565-72-4 | C_27_H_43_NO_4_ | Alkaloids |
| **170** | **Yibeinone A** | 1884621-00-4 | C_27_H_41_NO_5_ | Alkaloids |
| **171** | **Cycloposine** | 23185-94-6 | C_33_H_51_NO_7_ | Alkaloids |
| **172** | **Yibeinoside C** | 157536-48-6 | C_39_H_65_NO_12_ | Alkaloids |
| **173** | **Sevcoridinine** | 60269-68-3 | C_28_H_47_NO_2_ | Alkaloids |
| **174** | **Frititorine A** | NA | C_27_H_44_NO_3_ | Alkaloids |
| **175** | **Stenanzine** | 83133-08-8 | C_27_H_43_NO_3_ | Alkaloids |
| **176** | **Yibeinoside A** | 98985-24-1 | C_33_H_53_NO_7_ | Alkaloids |
| **177** | **Isopeimine** | 23496-43-7 | C_27_H_45_NO_3_ | Alkaloids |
| **178** | **Delavinone** | 96997-98-7 | C_27_H_43_NO_2_ | Alkaloids |
| **179** | **Ebeiedinone** | 25650-68-4 | C_27_H_43_NO_2_ | Alkaloids |
| **180** | **Delavine** | 98243-57-3 | C_27_H_45_NO_2_ | Alkaloids |
| **181** | **Peimisine** | 19773-24-1 | C_27_H_41_NO_3_ | Alkaloids |
| **182** | **Imperialine** | 61825-98-7 | C_27_H_43_NO_3_ | Alkaloids |

**Table. S3** Linearity and linear range of 9 control alkaloids.

| **Identification Compound** | **Linear Regression Equation** | **R2** | **Linear range (ng/ml)** |
| --- | --- | --- | --- |
| Edpetiline | y=2271.6x+234333 | 0.9994 | 141.00~2256.00 |
| Delavine | y=9604.8x+16125 | 0.9998 | 6.80~108.80 |
| Imperialine | y=3959.1x+487333 | 0.9970 | 129.00~2064.00 |
| Peimisine | y=5018.1x+7957.2 | 0.9991 | 6.91~110.60 |
| Yibeinoside A | y=5002x+87598 | 0.9997 | 35.88~574.00 |
| Verticinone | y=6091.7x+6336.1 | 0.9991 | 6.71~107.4 |
| Isopeimine | Y=6485.9X+12136 | 0.9994 | 9.06~145.00 |
| Delavinone | Y=6367.9X+773583 | 0.9976 | 138.75~2220.00 |
| Ebeiedinone | Y=7694.5X+232542 | 0.9993 | 65.25~1044.00 |

**Table. S4** Quantitative analysis of 9 alkaloids in BFP-TA.

| Identification Compound | CAS | Retention Time (min) | Content (%) |
| --- | --- | --- | --- |
| Edpetiline | 32685-93-1 | 3.989 | 3.998 |
| Delavine | 98243-57-3 | 4.202 | 0.107 |
| Imperialine | 61825-98-7 | 4.456 | 48.784 |
| Peimisine | 19773-24-1 | 4.500 | 1.590 |
| Yibeinoside A | 98985-24-1 | 4.565 | 0.223 |
| Verticinone | 18059-10-4 | 4.800 | 0.007 |
| Isopeimine | 23496-43-7 | 5.349 | 0.045 |
| Delavinone | 96997-98-7 | 5.400 | 7.861 |
| Ebeiedinone | 25650-68-4 | 5.780 | 1.199 |

**Table. S5** Differential genes in omics.

| No. | Gene_id | p-value | Type |
| --- | --- | --- | --- |
| 1 | Rap1gds1 | 4.805×10-3 | Proteomics-Transcriptomics |
| 2 | Vav2 | 4.358×10-2 | Transcriptomics |
| 3 | Racgap1 | 2.635×10-9 | Transcriptomics |
| 4 | PAK1 | 3.833×10-5 | \| Transcriptomics \| \| --- \| |

**Table. S6** Plasma-containing components of the administered groups (removal of plasma-containing components of the blank group).

| No. | Compound Name | CAS | Molecular Formula | Classification |
| --- | --- | --- | --- | --- |
| **1** | URATE | 69-93-2 | C_5_H_4_N_4_O_3_ | Organic acids |
| **2** | m-Hydroxycinnamic acid | [14755-02-3](https://commonchemistry.cas.org/detail?cas_rn=14755-02-3) | C_9_H_8_O_3_ | Organic acids |
| **3** | 3-Hydroxybutyric acid | 300-85-6 | C_4_H_8_O_3_ | Organic acids |
| **4** | ISOLEUCINE | [443-79-8](https://commonchemistry.cas.org/detail?cas_rn=443-79-8) | C_6_H_13_NO_2_ | Organic acids |
| **5** | Pantothenic acid | 79-83-4 | C_9_H_17_NO_5_ | Organic acids |
| **6** | Priverosaponin B 22-acetate | 144379-37-3 | C_56_H_90_O_25_ | Organic acids |
| **7** | 2-Hydroxy-4-methylpentanoate | NA | C_6_H_11_O_3_^-^ | Esters |
| **8** | 5-Hydroxy-3-indoleaceacetate | NA | C_11_H_11_NO_3_ | Esters |
| **9** | Baicalin | [21967-41-9](https://commonchemistry.cas.org/detail?cas_rn=21967-41-9) | C_21_H_18_O_11_ | Glycosides |
| **10** | 6-[[(3S,6aR,6bS,8aR,14bR)-9-hydroxy-4,4,6a,6b,8a,11,11,14b-octamethyl-1,2,3,4a,5,6,7,8,9,10,12,12a,14,14a-tetradecahydropicen-3-yl]oxy]-5-[4,5-dihydroxy-3-(3,4,5-trihydroxy-6-methyloxan-2-yl)oxyoxan-2-yl]oxy-3,4-dihydroxyoxane-2-carboxylic acid | NA | C_47_H_76_O_16_ | Organic acids |
| **11** | Taurocholic acid | 81-24-3 | C_26_H_45_NO_7_S | Organic acids |
| **12** | Indole-3-propionic acid | [830-96-6](https://commonchemistry.cas.org/detail?cas_rn=830-96-6) | C_11_H_11_NO_2_ | Organic acids |
| **13** | CHEMBL1616521 | NA | C_26_H_26_N_2_O_3_ | Alkaloids |
| **14** | Cholic acid | 81-25-4 | C_24_H_40_O_5_ | Organic acids |
| **15** | hyocholic acid | [547-75-1](https://commonchemistry.cas.org/detail?cas_rn=547-75-1) | C_24_H_40_O_5_ | Organic acids |
| **16** | Hyodeoxycholic acid | [83-49-8](https://commonchemistry.cas.org/detail?cas_rn=83-49-8) | C_24_H_40_O_4_ | Organic acids |
| **17** | C12-AS (TENTATIVE) | 151-41-7 | C_12_H_26_O_4_S | Organic acids |
| **18** | FA 18:1+2O | NA | C_18_H_34_O_4_ | Organic acids |
| **19** | Haloxyfop | [69806-34-4](https://commonchemistry.cas.org/detail?cas_rn=69806-34-4) | C_15_H_11_ClF_3_NO_4_ | Organic acids |
| **20** | LPC 18:3 | 62512-91-8 | C_26_H_48_NO_7_P | Alkaloids |
| **21** | Thymol-beta-D-glucoside | [20772-23-0](https://commonchemistry.cas.org/detail?cas_rn=20772-23-0) | C_16_H_24_O_6_ | Glycosides |
| **22** | FA 18:2+2O | NA | C_18_H_33_O_4_ | Esters |
| **23** | 8-HETE | [70968-93-3](https://commonchemistry.cas.org/detail?cas_rn=70968-93-3) | C_20_H_32_O_3_ | Organic acids |
| **24** | Octadecanedioic acid | [871-70-5](https://commonchemistry.cas.org/detail?cas_rn=871-70-5) | C_18_H_34_O_4_ | Organic acids |
| **25** | Canrenone | [976-71-6](https://commonchemistry.cas.org/detail?cas_rn=976-71-6) | C_22_H_28_O_3_ | Terpenes |
| **26** | Abietic acid | 514-10-3 | C_20_H_30_O_2_ | Organic acids |
| **27** | a-Linolenic acid | 463-40-1 | C_18_H_30_O_2_ | Organic acids |
| **28** | Arachidonic acid | 506-32-1 | C_20_H_32_O_2_ | Organic acids |
| **29** | (Z)-9,12,13-trihydroxyoctadec-15-enoic acid | NA | C_18_H_34_O_5_ | Organic acids |
| **30** | Palmitic acid | 57-10-3 | C_16_H_32_O_2_ | Organic acids |
| **31** | Oleic acid | [99148-48-8](https://commonchemistry.cas.org/detail?cas_rn=99148-48-8) | C_18_H_34_O_2_ | Organic acids |
| **32** | Cyclohexanecarboxylate | [3198-23-0](https://commonchemistry.cas.org/detail?cas_rn=3198-23-0) | C_7_H_11_O_2_^-^ | Esters |
| **33** | 1-(2,3,4-trihydroxyphenyl)ethanone | [528-21-2](https://commonchemistry.cas.org/detail?cas_rn=528-21-2) | C_8_H_8_O_4_ | Ketone |
| **34** | 3-Guanidinopropionic acid | [353-09-3](https://commonchemistry.cas.org/detail?cas_rn=353-09-3) | C_4_H_9_N_3_O_2_ | Organic acids |
| **35** | DEOXYCYTIDINE | [951-77-9](https://commonchemistry.cas.org/detail?cas_rn=951-77-9) | C_9_H_13_N_3_O_4_ | Glycosides |
| **36** | Benzoic acid | 65-85-0 | C_7_H_6_O_2_ | Organic acids |
| **37** | L-Methionine | 63-68-3 | C_5_H_11_NO_2_S | Organic acids |
| **38** | 4-Fluoroaniline | 371-40-4 | C_6_H_6_FN | Amines |
| **39** | 2'-Deoxycytidine | [951-77-9](https://commonchemistry.cas.org/detail?cas_rn=951-77-9) | C_9_H_13_N_3_O_4_ | Glycosides |
| **40** | 5-Methylcytosine | [554-01-8](https://commonchemistry.cas.org/detail?cas_rn=554-01-8) | C_5_H_7_N_3_O | Glycosides |
| **41** | Picolinamide | [1452-77-3](https://commonchemistry.cas.org/detail?cas_rn=1452-77-3) | C_6_H_6_N_2_O | Amines |
| **42** | 2-Phenylacetamide | [103-81-1](https://commonchemistry.cas.org/detail?cas_rn=103-81-1) | C_8_H_9_NO | Amines |
| **43** | Tyrosine | 60-18-4 | C_9_H_11_NO_3_ | Organic acids |
| **44** | Adenosine | 58-61-7 | C_10_H_13_N_5_O_4_ | Glycosides |
| **45** | ISOLEUCINE | [443-79-8](https://commonchemistry.cas.org/detail?cas_rn=443-79-8) | C_6_H_13_NO_2_ | Organic acids |
| **46** | Kynurenine | [343-65-7](https://commonchemistry.cas.org/detail?cas_rn=343-65-7) | C_10_H_12_N_2_O_3_ | Organic acids |
| **47** | L-Phenylalanine | 63-91-2 | C_9_H_11_NO_2_ | Organic acids |
| **48** | PANTOTHENATE | 79-83-4 | C_9_H_17_NO_5_ | Organic acids |
| **49** | Yohimbic Acid | [522-87-2](https://commonchemistry.cas.org/detail?cas_rn=522-87-2) | C_20_H_24_N_2_O_3_ | Alkaloids |
| **50** | Aminopyrine | 58-15-1 | C_13_H_17_N_3_O | Ketone |
| **51** | Pentaethylene glycol | 113894-92-1 | C_10_H_22_O_6_ | Alcohols |
| **52** | 2-Naphthylamine | 91-59-8 | C_10_H_9_N | Amines |
| **53** | glycyrrhetic acid | 471-53-4 | C_30_H_46_O_4_ | Terpenes |
| **54** | Ophiogenin-3-O-alpha-L-rhaMnopyranosyl-(1-->2)-beta-D-glucopyranoside | NA | C_39_H_62_O_14_ | Glycosides |
| **55** | 1,3-Diphenylguanidine | 102-06-7 | C_13_H_13_N_3_ | [Guanidine](https://pubchem.ncbi.nlm.nih.gov/compound/guanidine) |
| **56** | **Imperialine** | [61825-98-7](https://commonchemistry.cas.org/detail?cas_rn=61825-98-7) | C_27_H_43_NO_3_ | Alkaloids |
| **57** | Corticosterone | [50-22-6](https://commonchemistry.cas.org/detail?cas_rn=50-22-6) | C_21_H_30_O_4_ | Steroids |
| **58** | "(4R)-4-((3S,5R,6S,7S,9S,10R,13R,14S,17R)-3,6,7-trihydroxy-10,13-dimethylhexadecahydro-1H-cyclopenta[a]phenanthren-17-yl)pentanoic acid" | [63266-89-7](https://commonchemistry.cas.org/detail?cas_rn=63266-89-7) | C_24_H_40_O_6_ | Organic acids |
| **59** | MMV020120 | NA | C_23_H_34_N_2_O | Amines |
| **60** | DEOXYCHOLATE | 83-44-3 | C_24_H_40_O_4_ | Organic acids |
| **61** | chenodeoxycholic acid | 474-25-9 | C_24_H_40_O_4_ | Organic acids |
| **62** | Sphingosine-1-Phosphate (d18:1) | [26993-30-6](https://commonchemistry.cas.org/detail?cas_rn=26993-30-6) | C_18_H_38_NO_5_P | Phosphosphingolipid |
| **63** | NCGC00381425-01!8-hydroxy-8-(3-octyloxiran-2-yl)octanoic acid [IIN-based on: CCMSLIB00000846585] | NA | C_18_H_34_O_4_ | Organic acids |
| **64** | URSODEOXYCHOLATE | 128-13-2 | C_24_H_40_O_4_ | Organic acids |
| **65** | LAUROYLCARNITINE | [25518-54-1](https://commonchemistry.cas.org/detail?cas_rn=25518-54-1) | C_19_H_37_NO_4_ | Alkaloids |
| **66** | 1-palmitoyl-2-hydroxy-sn-glycero-3-phosphoethanolamine | 53862-35-4 | C_21_H_44_NO_7_P | Amines |
| **67** | Terbumeton | 33693-04-8 | C_10_H_19_N_5_O | Triazine |
| **68** | alpha-Naphthoflavone | [604-59-1](https://commonchemistry.cas.org/detail?cas_rn=604-59-1) | C_19_H_12_O_2_ | Flavonoids |
| **69** | Neoruscogenin | [17676-33-4](https://commonchemistry.cas.org/detail?cas_rn=17676-33-4) | C_27_H_40_O_4_ | Glycosides |
| **70** | Palmitoylcarnitine | [1935-18-8](https://commonchemistry.cas.org/detail?cas_rn=1935-18-8) | C_23_H_45_NO_4_ | Alkaloids |
| **71** | Isoreserpin | 50-55-5 | C_33_H_40_N_2_O_9_ | Alkaloids |
| **72** | PC(16:0/0:0) | [17364-16-8](https://commonchemistry.cas.org/detail?cas_rn=17364-16-8) | C_24_H_50_NO_7_P | Alkaloids |
| **73** | LysoPC(0:0/18:0) | [4421-58-3](https://commonchemistry.cas.org/detail?cas_rn=4421-58-3) | C_26_H_54_NO_7_P | Alkaloids |
